# Refractory Metal (Nb) Intermetallic Composites, High Entropy Alloys, Complex Concentrated Alloys and the Alloy Design Methodology NICE—Mise-en-scène † Patterns of Thought and Progress

**DOI:** 10.3390/ma14040989

**Published:** 2021-02-19

**Authors:** Panos Tsakiropoulos

**Affiliations:** Department of Materials Science and Engineering, Sir Robert Hadfield Building, The University of Sheffield, Mappin Street, Sheffield S1 3JD, UK; p.tsakiropoulos@sheffield.ac.uk

**Keywords:** Nb-silicide-based alloys, high entropy alloys, complex concentrated alloys, alloy design, alloy development, microstructures, oxidation, silicides

## Abstract

The paper reflects on the usefulness of the alloy design methodology NICE (Niobium Intermetallic Composite Elaboration) for the development of new Nb-containing metallic ultra-high-temperature materials (UHTMs), namely refractory metal (Nb) intermetallic composites (RM(Nb)ICs), refractory high entropy alloys (RHEAs) and refractory complex concentrated alloys (RCCAs), in which the same phases can be present, specifically bcc solid solution(s), M_5_Si_3_ silicide(s) and Laves phases. The reasons why a new alloy design methodology was sought and the foundations on which NICE was built are discussed. It is shown that the alloying behavior of RM(Nb)ICs, RHEAs and RCCAs can be described by the same parameters. The practicality of parameter maps inspired by NICE for describing/understanding the alloying behavior and properties of alloys and their phases is demonstrated. It is described how NICE helps the alloy developer to understand better the alloys s/he develops and what s/he can do and predict (calculate) with NICE. The paper expands on RM(Nb)ICs, RHEAs and RCCAs with B, Ge or Sn, the addition of which and the presence of A15 compounds is recommended in RHEAs and RCCAs to achieve a balance of properties.

## 1. Introduction

The targets given to the aerospace industry by international regulatory authorities regarding the environmental impact (noise, emissions) of aircraft in the future could be met with changes in airframes and aeroengines. The contribution of the latter to meet the targets is the reduction of CO_2_ by 30% and of the NO_x_ certification metric by 75% [1]. New materials are considered necessary to manufacture components that would enable critical parts of future aeroengines to operate at significantly higher temperatures (≥1850 °C) than those currently possible in high-pressure turbines (HPT) where coated and internally cooled single crystal Ni-based superalloy blades are used with turbine entry temperatures (TETs) not exceeding 1600 °C. In other words, there is a need for ultra-high-temperature materials (UHTMs) with capabilities beyond those of Ni-based superalloys [1,2,3].

The UHTMs must comply with specific property goals about creep, fracture toughness and oxidation. These goals are as follows: (a) for material density ρ = 7 g/cm^3^ the creep strength should be greater than 170 MPa at a creep rate of 2 × 10^−8^ s^−1^ at 1200 °C, (b) the fracture toughness of critical components should be ≥20 MPa√m and (c) the recession rate due to oxidation should be less than 0.25 μm/h at 1315 °C (to attain the oxidation life at 1315 °C of 2nd generation single crystal Ni-based superalloys at 1150 °C) [1,4]. The toughness goal requires the UHTMs to show some degree of metallic behavior to distinguish them from ceramic UHTMs. The latter is not considered in this paper.

Refractory metal intermetallic composites (RMICs), refractory (metal) high entropy alloys (RHEAs), and refractory (metal) complex concentrated alloys (RCCAs) proffer a paradigm for advanced metallic UHTMs to replace Ni-based superalloys [3]. RMICs include Nb-silicide-based alloys (RM(Nb)ICs) and Mo-silicide-based alloys (RM(Mo)ICs). The latter are not considered in this paper. The Nb-silicide-based alloys are referred to as RM(Nb)ICs from now on in this paper.

The paradigm about advanced metallic UHTMs is shared by the members of the international materials science and engineering community. Research on RMICs started in the 1990s and on RHEAs and RCCAs in 2010. The early research on RM(Nb)ICs originated in the USA, resulted in numerous patents, e.g., [5,6] and reviews of the processing, microstructure and properties of alloys, e.g., [2,4] and was followed with individual, national and international research programs in Europe, South America, Asia, the Far East and Russia. Research on RHEAs and RCCAs is nowadays ongoing in almost all parts of the world. Since the reviews on RM(Nb)ICs in early 2000, new research has addressed the alloying behavior and properties of RM(Nb)ICs and their phases. Since 2018, this research was published in [7,8,9,10,11] and was reviewed by the author in [1,3] and also resulted in a new patent [12]. Research on RHEAs and RCCAs was reviewed in 2018 in [13]. Lately, the RHEAs and RCCAs that were reviewed in [13] were compared with RM(Nb)ICs in [3]. Research on RM(Mo)ICs originated in the USA in the 1990s and resulted in patents, e.g., [14,15] and reviews, e.g., [16,17,18].

Alloys with refractory metal (RM) additions that satisfy the “standard definition” of HEAs as well as alloys where the higher or lower concentration of elements is above or below 35 and 5 at.%, i.e., the upper and lower limits in the “standard definition”, are designated, respectively RHEAs (elemental concentrations are in the range 35–5 at.%) and RCCAs (elemental concentrations can be >35 and <5 at.%) [13]. Some RM(Nb)ICs are also HEAs; others are also RHEAs or RCCAs, e.g., [1,3,19,20,21]. Not all RM(Nb)ICs are HEAs, RHEAs or RCCAs [1,3].

As many as twenty-three and twelve alloying elements have been reported, respectively in RM(Nb)ICs, and RHEAs or RCCAs, albeit not all in the same metallic UHTM, namely *Al*, B, C, Ce, *Cr*, Dy, Er, Fe, Ga, Ge, *Hf*, Ho, In, *Mo*, Nb, Si, Sn, *Ta*, *Ti*, V, *W*, Y, Zr, where underlined and in italics are the elements that can be found in the chemistry, respectively of RHEAs or RCCAs [1,3,13] and “state of the art” Ni-based superalloys for blade applications. Has realism been increasingly unrealistic in some research? Is Cervantes’s saying “paciencia y barajar” (“have patience, and keep shuffling the cards”) (keep shuffling the elements in our case) endorsed by researchers? How can one design/select metallic UHTMs to meet the property goals? How can one decide which elemental additions and concentrations are appropriate in a metallic UHTM? Are all (or which of) the aforementioned elements essential to meet the property goals? Which of these elements are the key ones to achieve a balance of properties? Are the challenges in alloy design/selection that arise from (i) the lack (and often disagreement) of thermodynamic data for key binary and ternary systems [1,3], (ii) the sensitivity of elemental additions and alloys to interstitial contamination [3,13] and (iii) the pest oxidation phenomenon [1,3], and the scale-up of alloy production and confirmation/reproducibility of results produced from small buttons [3] overlooked (disregarded, ignored)?

The RM(Nb)ICs are multiphase alloys with microstructures that consist of intermetallic(s) (M_3_Si and M_5_Si_3_ silicides (M = RM and/or transition metal (TM)), C14 Laves phase and A15 compounds) with/without bcc solid solution(s) [1,3]. The RHEAs and RCCAs can be (a) single-phase bcc solid solution, (b) two (or more?) solid solutions (of same or different crystal structure), (c) mixture of solid solution(s) and intermetallic(s) (Laves and M_5_Si_3_ silicide(s?)) [13]. The early research associated HEAs, RHEAs, and RCCAs with (a), the subsequent research revealed that the microstructure of some as produced single-phase solid solution alloys can change to (b) or (c) at elevated temperatures or that RHEAs and RCCAs can be produced with (b) or (c). Some of the aforementioned elements are added (i) in RM(Nb)ICs to suppress pest oxidation, increase oxidation resistance at high temperatures and suppress scale spallation, increase toughness, increase room, intermediate and high-temperature strength and decrease creep deformation [1,3,19] and (ii) in RHEAs and RCCAs to improve oxidation or room and high-temperature strength [3,13] (currently there is no data about the toughness and creep properties of RHEAs and RCCAs).

Einstein asserted that scientific research requires imagination, which often takes visual forms [22] that can become the starting points for some work. RM(Nb)ICs, RHEAs and RCCAs share the same alloying additions, and the parameters δ, Δχ, VEC, ΔH_mix_, ΔS_mix_ and Ω (= T_m_ΔS_mix_/∣ΔH_mix_∣) (see Appendix A) describe their alloying behavior as they do for HEAs [1,3,8,13,23]. The values of these parameters for bcc solid solutions, metallic UHTMs and HEAs overlap (Table 1). There are visual equivalents that can be used to explain the alloying behavior of alloys and their phases. The alloy design methodology NICE [1] (Niobium Intermetallic Composite Elaboration) has made it possible to construct such visual forms, namely maps based on the aforementioned parameters. Indeed, RM(Nb)ICs, RHEAs and RCCAs not only share common alloying elements, but they also share “common ground in parameter maps” [3,24,25]. Maps of aforementioned parameters, for example δ versus ΔH_mix_ or ΔS_mix_, have been produced in studies of phase stability in HEAs and to compare the latter with bulk metallic glasses (BMGs).

In this paper, I reflect on the utility of NICE to design and develop new metallic UHTMs with Nb one of the alloying additions, drawing on my recent publications [1,3,7,8,9,10,11] and, where it is appropriate, on papers that I have co-authored with members of the research group. I discuss (i) why a new alloy design methodology was sought for metallic UHTMs and the foundations on which NICE was built, (ii) how NICE helps one to understand better the alloys s/he develops, and (iii) what one can do and predict (calculate) with NICE. In this paper, I do not cover the processing, oxidation and fracture toughness of metallic UHTMs, about which the interested reader could refer to [3]. In addition, I do not compare the properties of RM(Nb)ICs with those of the RHEAs and RCCAs that were reviewed by Senkov et al. in [13]; an interested reader could consult the ref [3]. However, in this paper, I expand on RM(Nb)ICs with B, Ge or Sn additions, some of which are also RHEAs and RCCAs [19], and in doing so, I confirm that the above elements are key additions in RHEAs and RCCAs to achieve a balance of properties.

In this paper, my approach to the subject matter is to provide a brief coverage/review of the alloying of RM(Nb)ICs (discussed in the refs [7,8,9,10,11] that dealt closely with issues that pertain to the alloys and their phases) and to show how my ideas that were instigated from these studies, despite their having been conceived and organized separately over many years, converged and were unified in NICE [1] to give a perspicuous and straightforward account of a new alloy design methodology that is useful both for RM(Nb)ICs and for RHEAs and RCCAs [1,3,19,20,21,24,25]. I use this opportunity to draw attention to RM(Nb)ICs and RCCAs with B, Ge or Sn additions and expand on the data presented in [1,3,7,8,9,10,11] and, most recently, in [19,26]. The structure of the paper is as follows: First, we return to the alloying behavior of metallic UHTMs and their phases, then an overview of properties of alloys and phases and of relationships between alloys and their phases is given, and finally, the alloy design/selection methodology NICE is revisited.

## 2. Alloying Behavior of RM(Nb)ICs, RHEAs and RCCAs and Their Phases

**Figure 1:**
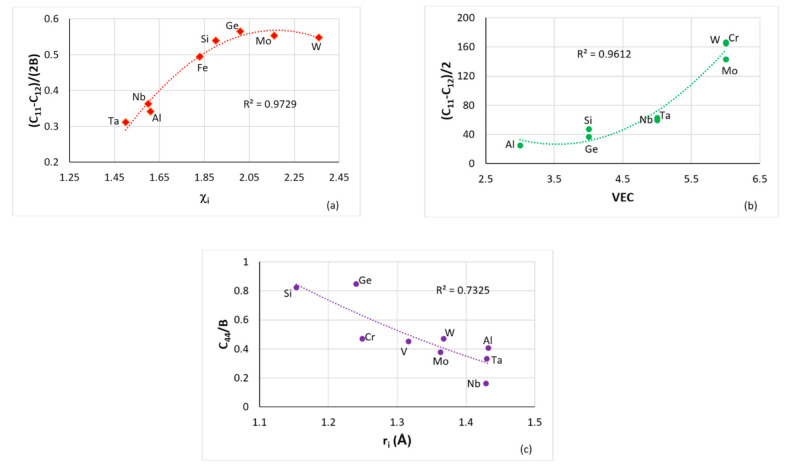

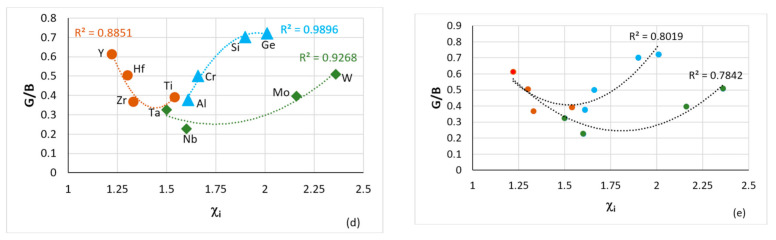
(**a**–**c**) data for cubic symmetry elements in RM(Nb)ICs, (**a**) plot of [C_11_-C_12_]/[2B] versus Pauling electronegativity χ_i_ for Al, Fe, Ge, Mo, Nb, Si, Ta and W, (**b**) plot of (C_11_-C_12_)/2 versus VEC for Al, Cr, Ge, Mo, Nb, Si, Ta and W and (**c**) plot of C_44_/B for Al, Cr, Ge, Mo, Nb, Si, Ta, V and W. (d) Plot of G/B versus Pauling electronegativity for cubic and hexagonal symmetry elements, circles for Hf, Ti, Y, Zr, triangles for Al, Cr, Ge, Si and diamonds for Mo, Nb, Ta, W. (**e**) is the same as (**d**) and shows one group containing Y, Hf, Zr, Ti, Al, Cr, Si, Ge (R^2^ = 0.8019) and another containing Y, Hf, Zr, Ti, Ta, Nb, Mo, W (R^2^ = 0.7842). Note that with the exception of Fe in (**a**), all other elements in (**a**–**d**) are used in RHEAs and RCCAs (for Ge see text) and that Al, Cr, Ge, Hf, Si, Ti improve the oxidation of RM(Nb)ICs [1]. In (a) R^2^ = 0.9726 if Fe were to be excluded.

Alloying elements mentioned in the previous section fall in different groups in plots (a) of diffusivity versus atomic size r_i_ or Pauling electronegativity χ_i_ [7], (b) of functions of elastic constants C_ij_ (e.g., the Zener anisotropy constant) versus r_i_ or χ_i_ or the number of valence electrons per atom filled into the valence band (VEC) [8] (see also Figure 1) and (c) of Young’s modulus versus r_i_ or χ_i_ [8]. Remarkably, in (a) and (c), where B is included in the data, the B always belongs in specific groups with/without the elements Al, Cr, Ge, Hf, Si, Sn, Ti and with/without RMs [7,8]. The elements Al, B, Cr, Ge, Hf, Si, Sn, Ti are key additions for improving the oxidation resistance of RM(Nb)ICs, meaning suppressing pest oxidation and improving oxidation resistance at high temperatures [1,3], and suppressing scale spallation [1,3,19,20,21,25,26]. To the author’s knowledge, additions of B, Ge or Sn have not been reported in RHEAs, and RCCAs studied to date and were not reported for the RCCAs reviewed in [13], but they have been used only in RM(Nb)ICs some of which are also RHEAs or RCCAs, e.g., [3,19,20,21,25]. We return to these three elements as alloying addition(s) in metallic UHTMs below in this section and in the following sections.

Figure 1 shows plots of elastic constants C_ij_, shear modulus G and bulk modulus B versus atomic size r_i_, Pauling electronegativity χ_i_ and VEC of cubic (Figure 1a,d) and hexagonal (Figure 1d) symmetry elements. Note that Fe currently is not used as elemental addition in RHEAs or RCCAs [13] and that in Figure 1a the group and R^2^ value do not significantly change if Fe were to be excluded. In Figure 1d, note (i) that the elements Hf, Nb, Mo, W or Hf, Ti, Al, Cr, Si, Ge could be grouped together (see Figure 1e), (ii) that the latter six elements improve oxidation resistance in RM(Nb)ICs [1] as well as in RM(Nb)ICs that are also RCCAs [3,19,26,27] and suppress pest oxidation [27], (iii) that the former four elements are key additions (a) in RM(Nb)ICs for bcc solid solution strength and alloy strength [3] and for meeting the creep goal [3,12] and (b) in RCCAs for enhanced strength [3,13] and for improved oxidation resistance when added together with Al, Cr, Ti and Si [13]. Recently, our research group suggested that RM(Nb)ICs, RHEAs and RCCAs of the Nb-Mo-W-Ti-Cr-Hf-Al-Ge-Si-Sn system are worthy of development owing to their promise to meet property goals and/or offer a balance of properties [19]. Such metallic UHTMs could be protected by environmental coatings (ECs) of the bond coat (BC)/thermally grown oxide (TGO)/top coat (TC) type with BC consisting of αAl_2_O_3_ scale forming HEAs of the Nb-Ti-Si-Al-Hf system [19,20,21] (compatible with the metallic UHTM substrate) with/without αAl_2_O_3_ or Cr_2_O_3_ and SiO_2_ scale forming Si-rich intermetallic alloys of the Al-Cr-Fe-Nb-Si-Ti system [28] that are also compatible with the aforementioned HEA BC [28,29]. We return to ECs for metallic UHTMs in Section 5.

**Figure 2:**
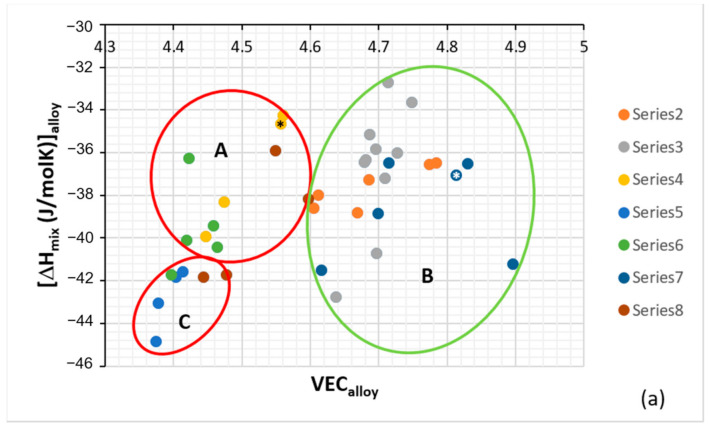

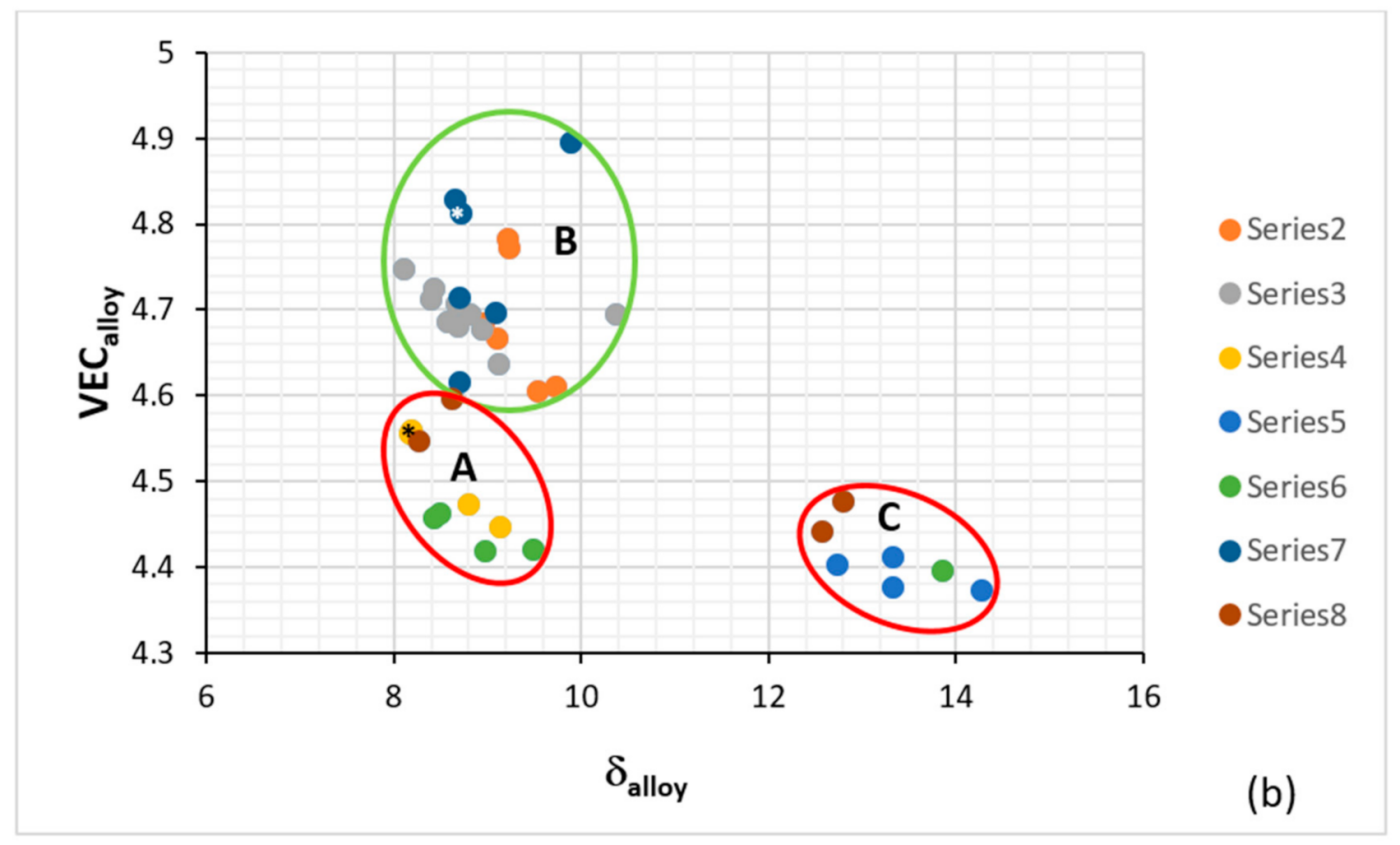
Plots of ΔH_mix_ versus VEC (**a**) and VEC versus δ (**b**). The alloys KZ5 and YG8 are in areas A and B. Alloys with Boron are only in area C. Group A alloys based on KZ5 with Nb_ss_ and Nb_5_Si_3_ with/out Laves phase, Group B alloys based on YG8 with Nb_ss_ and Nb_5_Si_3_, Group C alloys based on KZ series alloys [31,33] with B addition. There are no boron-containing alloys in areas A and B. Alloys with RMs, Al, Cr, Sn and Ge are in all three areas. The alloys KZ5 and YG8 are indicated by asterisks. For the alloys of series 2 to 8, see Table 1 in [8], where also the values of the parameters Δχ, δ, VEC, ΔH_mix_, ΔS_mix_, Ω are given.

Metallurgists have used atomic size, electronegativity, the heat of mixing ΔH_mix_, the entropy of mixing ΔS_mix_ and VEC to study alloys prior to HEAs, for example, for the study of rapidly solidified (RS) alloys and metallic glasses. The role of the researcher is not so much to collect and analyze data as to bring “trained judgment” to bear on the data. One often needs to capture similarities as well as differences (s) in his/her data. Consideration (i) of the alloying behavior of RM(Nb)ICs using the same parameters, namely Δχ (based on Pauling electronegativity), δ (based on atomic size), VEC, ΔH_mix_, ΔS_mix_, Ω that are used (1) for the study of HEAs [23] and (2) to compare the latter with BMGs, e.g., [30], and (ii) of the (a), (b) and (c) in the first paragraph of this section and Figure 1, necessitated scrutiny of our work from a new perspective. We did not just consider “what do these parameters refer to?” but “how do we use these parameters?” what roles may possibly have in alloy design and how these roles change, overlap, and can be shown/demonstrated. This experience confronted me with new ways of interpreting our work that exceeded the limits of what can be perceived and visualized in familiar terms and made possible (A) the classification of the RM(Nb)ICs (and RCCAs) in groups, (B) new ways of presenting data (Figure 2, also see [8] and Figure 19 in [3]), and (C) the comparison of RM(Nb)ICs with RCCAs [3] and with HEAs and metallic glasses (Table 1). To sift and assess raw data required some form of visual representation and thus an element of informed subjectivity. After assessing the data, it was decided to separate all the alloys with boron addition, and in doing so, it was discovered that these alloys formed a separate group (group C) in parameter maps, see Figure 2. This then steered scrutiny of the data of the boron-free alloys, and in doing so, it was discovered that alloys that meet or have the potential to meet the creep property goal could form a separate group (group B) in parameter maps, see Figure 2. The grouping of alloys shown in Figure 2 considered RM(Nb)ICs based on the alloys KZ5, and YG8 with nominal compositions, respectively Nb-24Ti-18Si-5Al-5Cr [31] and Nb-20Si-5Hf-5Mo-3 W [32], of which the former meets the toughness goal, and the latter is close to meeting the creep goal [3,12], with additions of other TMs, RMs and simple metal (SM) and metalloid (Met) elements.

Remarkably, (a) groups A and B in Figure 2 fall within the areas occupied by RCCAs in similar maps, for example, the Δχ versus δ map in Figure 19 in [3] (otherwise stated, the RHEAs and RCCAs include some RM(Nb)ICs), (b) oxidation-resistant metallic UHTMs can be found in all three groups A, B and C in Figure 2 and (c) some of the RM(Nb)ICs in group C are also RHEAs and RCCAs (see below and following sections) with exceptional oxidation resistance [3]. In essence, (i) the alloying behavior of RM(Nb)ICs, RHEAs and RCCAs can be presented in Δχ versus δ, ΔH_mix_ versus VEC and VEC versus δ maps and (ii) there are RHEAs and RCCAs with B, Ge or Sn addition that are also RM(Nb)ICs [1,3,19] (or to put it in another way, some RM(Nb)ICs with B, Ge or Sn addition can also be RHEAS and RCCAs).

**Figure 3:**
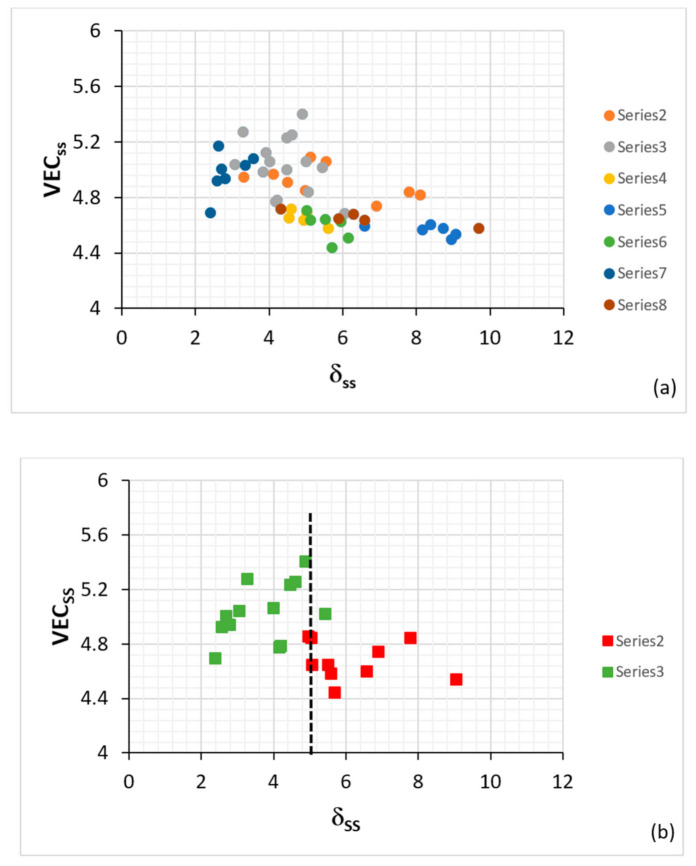
Valence electron concentration (VEC) versus δ maps for the bcc Nb_ss_ in Nb-silicide-based alloys, (**a**) all data and (**b**) data for Si-free Nb_ss_ and for Nb_ss_ rich in Ti. In (**a**) the series 2 data are for Nb_ss_ with RMs and Sn and no Al, the series 3 is for Nb_ss_ with RMs, Ge and Sn and with/out Al and Cr, the series 4 is for Nb_ss_ with Al, Cr, with/out Hf, no RMs and no B, Ge, Sn, the series 5 is for Nb_ss_ with Al, B, Cr with/out Hf, the series 6 is for Nb_ss_ with Al, Cr, with/out Ge or Sn or B, the series 7 is for Nb_ss_ with RMs, with/out Al and with no B, Cr, Ge, Sn and the series 8 is for Nb_ss_ with TMs, Al with/out RMs, B or Sn and no Ge. In (**b**) the series 2 is for Nb_ss_ rich in Ti, and series 3 is for Si-free Nb_ss_. RM = Mo,Ta,W, TM = Cr,Hf,Ti. For the chemical composition of the solid solutions, see Table 1 in [7]. For dashed line in (b), see text. Note that the solid solutions included in this figure belong to the alloys for which data are given in Figure 2.

**Figure 4:**
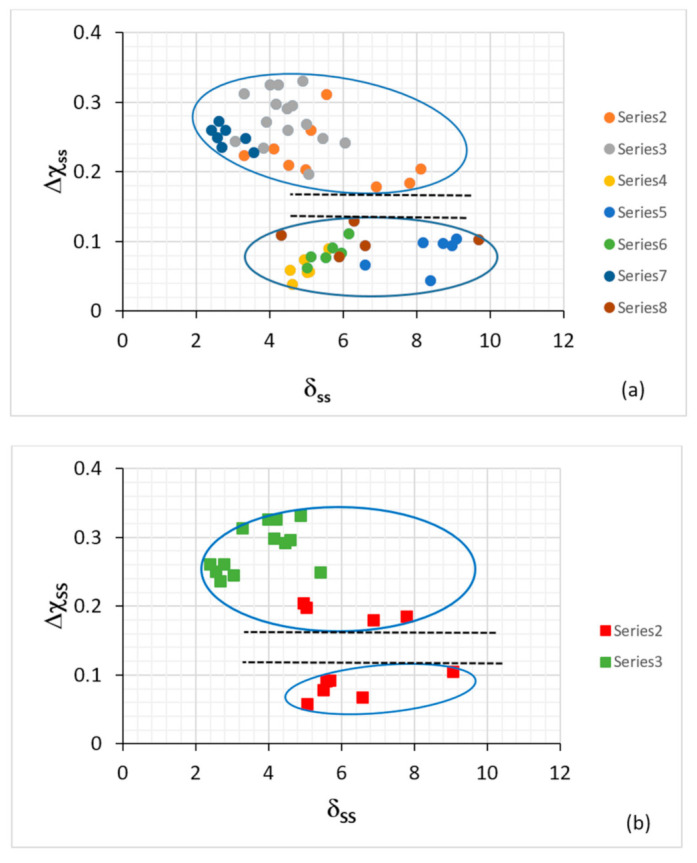
Δχ versus delta (δ) maps for Nb_ss_ in Nb-silicide-based alloys, (**a**) all data and (**b**) data only for Si-free Nb_ss_ and Nb_ss_ rich in Ti. In (**a**) the series 2 data are for Nb_ss_ with RMs and Sn and no Al, the series 3 is for Nb_ss_ with RMs, Ge and Sn and with/out Al and Cr, the series 4 is for Nb_ss_ with Al, Cr, with/out Hf, no RMs and no B, Ge, Sn, the series 5 is for Nb_ss_ with Al, B, Cr with/out Hf, the series 6 is for Nb_ss_ with Al, Cr, with/out Ge or Sn or B, the series 7 is for Nb_ss_ with RMs, with/out Al and with no B, Cr, Ge, Sn and the series 8 is for Nb_ss_ with TMs, Al with/out RMs, B or Sn and no Ge. In (**b**) the series 2 is for Nb_ss_ rich in Ti, and series 3 is for Si-free Nb_ss_. RM = Mo,Ta,W, TM = Cr,Hf,Ti. For the chemical composition of the solid solutions, see Table 1 in [7]. For dashed lines and ellipses, see text. Note that the solid solutions included in this figure belong to the alloys for which data are given in Figure 2.

If the alloying of metallic UHTMs can be described using the parameters Δχ, δ, VEC, ΔH_mix_, ΔS_mix_, Ω, why the phases that are present in the microstructures of these materials were not studied using these parameters before the publication of [7,9,10,11]? A reasonable answer is that it is partly a matter of where researchers “point their instruments” to collect data and partly a matter of what they instructed their instruments to find for them. In our research group, we used reliable quantitative chemical analysis data (EPMA and EDS with standards) for RM(Nb)ICs and their phases and created maps for their bcc solid solutions (Figure 3) [7,8,9,10,11]. In RM(Nb)ICs, three types of bcc Nb solid solution (Nb_ss_) can form, namely “normal” Nb_ss_, Si-free Nb_ss_ and Ti-rich Nb_ss_ [7]. In RCCAs, one or more solid solutions can form [13]. The Ti-rich Nb_ss_ is observed in as-cast (AC) RM(Nb)ICs. Figure 3a shows the VEC_ss_ versus δ_ss_ map for all the bcc solid solutions in AC and heat-treated (HT) RM(Nb)ICs (the chemical compositions of the solid solutions in this figure were given in table 1 in [7]) and would suggest that VEC_ss_ decreases as δ_ss_ increases, like the parameter (ΔH_mix_)_ss_ in the (ΔH_mix_)_ss_ versus δ_ss_ map [3,7]. Figure 3b shows that the Si-free Nb_ss_ has δ_ss_ less than approximately 5, which is also the case in the (ΔH_mix_)_ss_ versus δ_ss_ map for RM(Nb)ICs [7], whereas the solid solutions of RCCAs have 4 < δ < 6 [3]. The parameter Δχ_ss_ would suggest that there exist no bcc Nb solid solutions in RM(Nb)ICs for 0.13 < Δχ_ss_ < 0.18 (Figure 4a), and that the Si-free Nb_ss_ has Δχ > 0.24 (Figure 4b). The same gap exists in the case of solid solution RCCAs [3], and there is also a gap in the Δχ values of eutectics with Nb_ss_ and Nb_5_Si_3_ [11]. Are these gaps “real” or just “an upshot of the available experimental data”? My response to this question is as follows: “There is actually a certain value in not finding data in some part of a map, in other words in having gaps in parameter values. They are some of those areas where the absence of evidence currently is evidence”.

**Figure 5:**
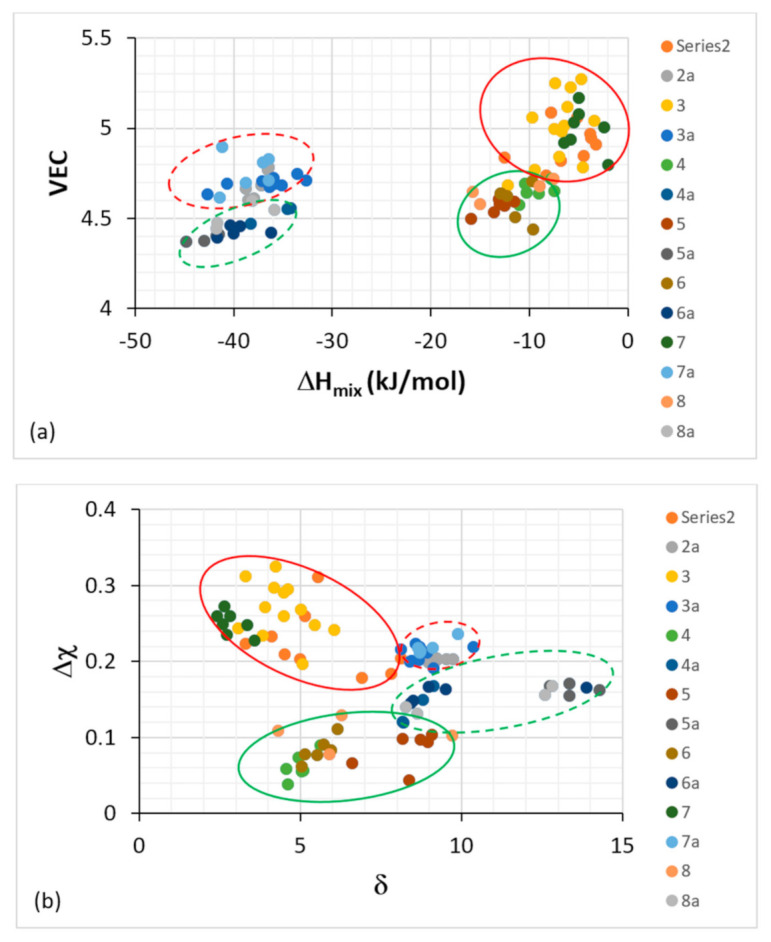
Plot of VEC versus ΔH_mix_ (**a**) and Δχ versus δ (**b**) for Nb-silicide-based alloys and their Nb_ss_ solid solutions. The dashed ellipses are for the alloys and the full ones for the Nb_ss_ solid solutions. The red ellipses are for series 2, 2a, 3, 3a, 7, 7a, where the alloying elements are refractory metals (RMs), Al, Cr, Ge, Sn, but no boron. The green ellipses are for series 4, 4a, 5, 5a, 6, 6a, 8, 8a, where the alloying elements are RMs, Ge, Hf, Sn and boron. The series 2 to 8 data are for the three types of bcc Nb_ss_ solid solutions in Table 1 in [7], and the series 2a to 8a data are for Nb silicide-based alloys in Table 1 in [8]. The series 2 and 2a data are for alloys with RMs, TMs, and Sn, but no Al, the series 3 and 3a data are for alloys with RMs, TMs, Ge and Sn and with/without Al and Cr, the series 4 and 4a data are for alloys with TMs, Al, with/without Hf, no RMs and no B, Ge, Sn, the series 5 and 5a data are for alloys with TMs, Al and B, with/without Hf, the series 6 and 6a data are for alloys with TMs, Al and with/without B, Ge, Hf and Sn, the series 7 and 7a data are for alloys with RMs, TMs with/without Al and with no B, Ge, Sn and the series 8 and 8a data are for alloys with TMs, Al with/without RMs, B or Sn and no Ge.

The alloys and their solid solutions can also be presented together in maps of the aforementioned parameters [8], and they can be separated into distinctly different groups in VEC versus ΔH_mix_, Δχ versus δ (Figure 5), and Δχ versus ΔH_mix_ and Δχ versus Ω maps [8] in which the B-containing alloys and solid solutions occupy separate groups. Basically, (i) the alloying behavior of solid solutions in RM(Nb)ICs, and solid solution RHEAs and RCCAs can be presented in Δχ versus δ, Δχ versus VEC, ΔH_mix_ versus δ and VEC versus δ maps [1,3] and (ii) the data includes solid solutions in RM(Nb)ICs with B, Ge or Sn addition that are (the alloys) also RHEAs and RCCAs [1,3,19,26].

**Figure 6:**
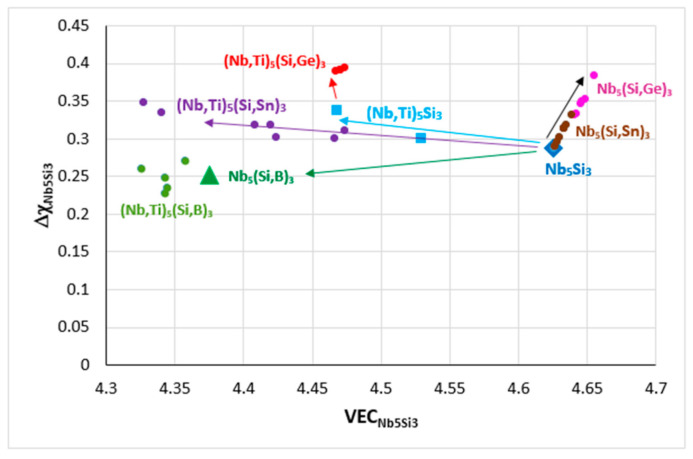
Δχ versus VEC map of Nb_5_Si_3_. Data for unalloyed Nb_5_Si_3_ (diamond), silicide where Si is substituted by Sn (brown circles) or Ge (pink circles) or B (triangle), silicide where Nb is substituted by Ti (squares) and silicide where Nb is substituted by Ti and Si by B (green circles), Sn (purple circles), Ge (red circles). Data for Nb_5_Si_3,_ where Nb is substituted by TM and RM additions and Si by simple metal and metalloid elements (Al, B, Ge, Sn), can be found in [9].

**Figure 7:**
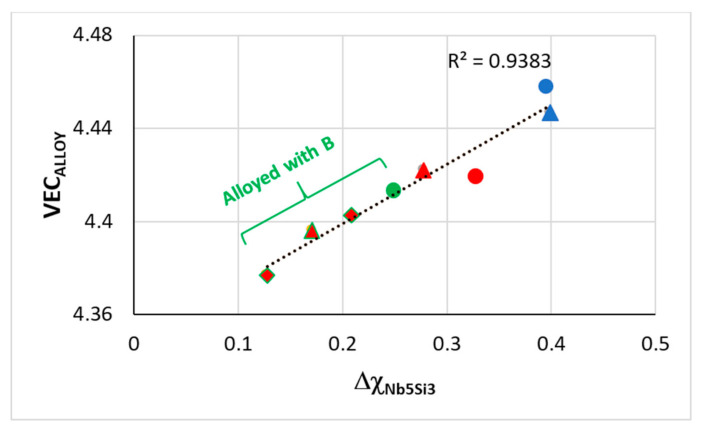
VEC versus Δχ map for RM(Nb)ICs based on the alloy KZ5 with additions of B, Ge, Hf, Sn. RM(Nb)ICs that are also RCCAs are shown with red color. For the linear fit of all data, R^2^ = 0.9383. Green and red-green data: Diamonds for KZ5 + Hf + B (RM(Nb)IC-RCCA), triangle for KZ5 + B + Sn (RM(Nb)IC-RCCA), green circle for KZ5 + B, red triangle for KZ5 + Hf + Sn (JG6-RM(Nb)IC-RCCA), red circle for KZ5 + Ge + Hf (ZF9-RM(Nb)IC/RCCA). Blue data: circle for KZ5 + Ge (ZF6) and triangle for KZ5 + Hf (JN1). Data about the densities and room temperature strength of these alloys is given in [3]. Nominal compositions (at.%) of B-containing RM(Nb)ICs-RCCAs 37Nb-24Ti-18Si-6B-5Al-5Cr-5Hf, 39Nb-24Ti-18Si-6B-5Al-4Cr-4Sn. For nominal compositions of other alloys, see Appendix A Table A1.

The alloying behavior of Nb_5_Si_3_ is shown in Figure 6. The substitution of Si by Sn in Nb_5_(Si,Sn)_3_ increases both VEC and Δχ, these parameters increase further when Si is substituted by Ge in Nb_5_(Si,Ge)_3_ (shown by the black arrow in Figure 6), whereas the substitution of Si by B in Nb_5_(Si,B)_3_ has the opposite effect, causing a significant decrease of VEC and a small decrease of Δχ (shown by the green arrow). The substitution of Nb by Ti in the silicide decreases VEC and slightly increases Δχ in (Nb,Ti)_5_Si_3_ (blue arrow) and the changes of VEC and Δχ are enhanced in (Nb,Ti)_5_(Si,Ge)_3_ and (Nb,Ti)_5_(Si,Sn)_3_ (purple and red arrows, respectively), whereas, for the Nb_5_Si_3_ alloyed with B and Ti, the VEC is decreased further compared with the Nb_5_(Si,B)_3_. Similarly, with the RM(Nb)ICs alloyed with B, which, as we have seen, occupy a separate area (C) in parameter maps (Figure 2), the alloyed 5–3 silicide with the addition of B also occupies a distinct, separate area in Figure 6. The effect of B on the alloying behavior of Nb_5_Si_3_ is also shown in Figure 7, where the data are for RM(Nb)ICs based on KZ5 (Nb-24Ti-18Si-5Al-5Cr, nominal composition [31]) with additions of B, Ge, Hf or Sn. Note (a) the remarkable linear fit of data and (b) that the diamond data points correspond to RM(Nb)ICs that are also RCCAs.

**Figure 8:**
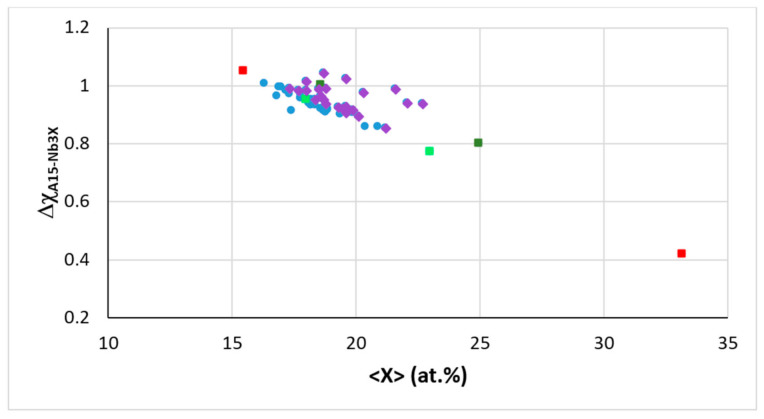
Δχ versus <X> = Al + Ge + Si + Sn in A15-Nb_3_X where Nb is substituted by Cr, Fe, Hf, Mo, Ti or W and X = Al, Ge, Si or Sn. Squares, red Nb_3_Sn (Sn = 15.5 and 33.2 at.%), dark green Nb_3_Al (Al = 18.6 and 25 at.%) and light green Nb_3_Ge (Ge = 18 and 23 at.%). Diamonds for Nb substituted by Cr, Hf, Mo, Ti or W and X = Al, Ge, Si or Sn, filled circles for Nb substituted by Cr, Fe, Hf, or Ti and X = Al, Si or Sn. Linear fit of all data gives R^2^ = 0.7484.

**Figure 9:**
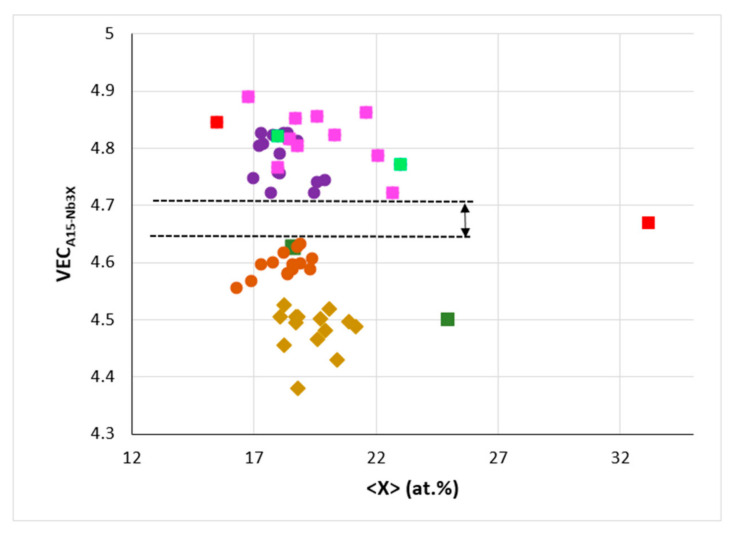
VEC versus <X> = Al + Ge + Si + Sn in A15-Nb_3_X, where Nb is substituted by Cr, Fe, Hf, Mo, Ti or W and X = Al, Ge, Si or Sn. Squares, red Nb_3_Sn (Sn = 15.5 and 33.2 at.%), dark green Nb_3_Al (Al = 18.6 and 25 at.%) and light green Nb_3_Ge (Ge = 18 and 23 at.%), pink for Nb substituted by Cr, Hf, Mo, Ti or W and X = Al, Ge, Si or Sn. Diamonds for Nb substituted by Cr, Hf or Ti and X = Al, Si or Sn, Filled circles, purple for Nb substituted by Cr or Hf and X = Al, Si or Sn, orange for Nb substituted by Cr, Fe, Hf or Ti, and X = Al, Si or Sn. Note the gap of VEC values (from 4.628 to 4.721) for alloyed Nb_3_X, and that data point for Sn rich Nb_3_Sn falls in this gap.

The alloying behavior of the C14-NbCr_2_ Laves phase and A15-Nb_3_X compounds (X = Al, Ge, Si, Sn) was discussed in [10] and of eutectics with Nb_ss_ and Nb_5_Si_3_ in [11]. In the refs [10,11], maps of the aforementioned parameters can be found. The data in Figure 8 shows that the parameter Δχ of A15-Nb_3_X (i) decreases with increasing <X> = Al + Ge + Si + Sn, (ii) is in the range 0.855 to 1.04 for the alloyed Nb_3_X where Nb is substituted by Cr, Fe, Hf, Mo, Ti or W and (iii) does not deviate significantly from the trend established by the data for the unalloyed Nb_3_Al, Nb_3_Ge, Nb_3_Sn. A similar decrease of the parameter Δχ of C14-NbCr_2_ occurs with alloying, but in the case of the Laves phase, the substitution of Nb and Cr by alloying additions shifts the data to higher Δχ values compared with the unalloyed Laves phase [10]. Remarkably, there is a gap (from 4.628 to 4.721) in the values of the parameter VEC of alloyed A15-Nb_3_X even though the data point for Sn rich Nb_3_Sn falls in this gap (Figure 9). The same gap was shown in the Δχ versus VEC map in [10]. Essentially, the alloying behavior of the intermetallics that can be stable in the microstructures of RM(Nb)ICs, some of which are also RHEAs or RCCAs, can be described using maps of the aforementioned parameters. Unfortunately, there is no data for the Laves phases and M_5_Si_3_ silicides that are observed in the RHEAs and RCCAs that were reviewed in [13] to enable the construction of maps and to compare them with those of the intermetallic compounds in RM(Nb)ICs.

**Figure 10:**
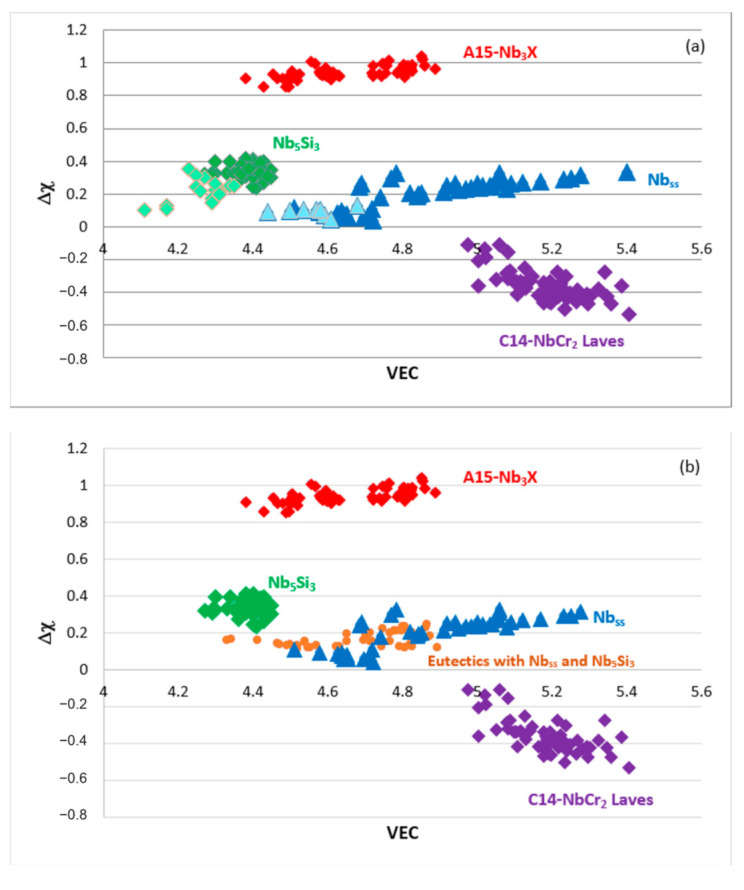
(**a**,**b**) show maps of Δχ versus VEC. In (**a**), the data are for the Nb_ss_ (blue triangles), Nb_5_Si_3_ (green diamonds), C14-NbCr_2_ Laves (purple diamonds) and A15-Nb_3_X (red diamonds) phases, and the boron-containing Nb_ss_ and Nb_5_Si_3_ are shown in light blue and light green. In (**b**), the data are for the Nb_ss_ (blue triangles), Nb_5_Si_3_ (green diamonds), eutectics with Nb_ss_ and Nb_5_Si_3_ (orange circles), C14-NbCr_2_ Laves (purple diamonds) and A15-Nb_3_X (red diamonds) phases. The data for the bcc solid solution RCCAs studied by Senkov et al. [13], and HEA Nb_ss_ and HEA Nb_ss_ and Nb_5_Si_3_ eutectics in RM(Nb)ICs that satisfy the “standard definition” of HEAs fall in the area of the solid solution and eutectics data in (**b**), as shown in Figure 16 in [3]. Note that both parts of this figure are the same as those in Figure 5 in [1], in which by mistake, the labels for the A15-Nb_3_X and C14-NbCr_2_ phases were swapped.

The Δχ versus VEC maps of the phases in RM(Nb)ICs are shown in Figure 10. These and the Δχ versus VEC map in Figure 16 in [3] are the “master maps” of metallic UHTMs (excluding RM(Mo)ICs). Note (a) that Figure 10 is a correction of Figure 5 in [1], in which by mistake, the labels for the A15-Nb_3_X and C14-NbCr_2_ phases were swapped, (b) that the B-containing Nb_ss_ and Nb_5_Si_3_ “sit” in the left-hand side of the map in Figure 10a, (c) that eutectics with Nb_ss_ and Nb_5_Si_3_ are positioned in the area between approximately 0.12 < Δχ < 0.25 and 4.3 < VEC < 4.9 that is also occupied by the bcc solid solution (Figure 10b), (d) that the data for the bcc solid solution RCCAs studied by Senkov et al. [13] and the HEA Nb_ss_ and HEA eutectics with Nb_ss_ and Nb_5_Si_3_ in RM(Nb)ICs that satisfy the standard definition of HEAs fall in the area of the Nb_ss_ and eutectics with Nb_ss_ and Nb_5_Si_3_ in Figure 10b, as discussed in [3].

The parameters Δχ, δ, VEC, ΔH_mix_, ΔS_mix_, Ω can be seen as being nothing more than guidelines to make alloys. This they do with a certain monotonous consistency. In this sense, they are like the keys of the piano, each playing a single note, but combine them (in NICE as we discuss below) as you would combine piano keys, and you can create “melodies of infinite variety”. Put all these parameters together (in NICE as we discuss below), and you have “the great symphony of RM(Nb)ICs, HEAs, RHEAs, RCCAs”.

## 3. Alloying Behavior of RM(Nb)ICs, RHEAs and RCCAs and Properties of Alloys and Phases

**Figure 11:**
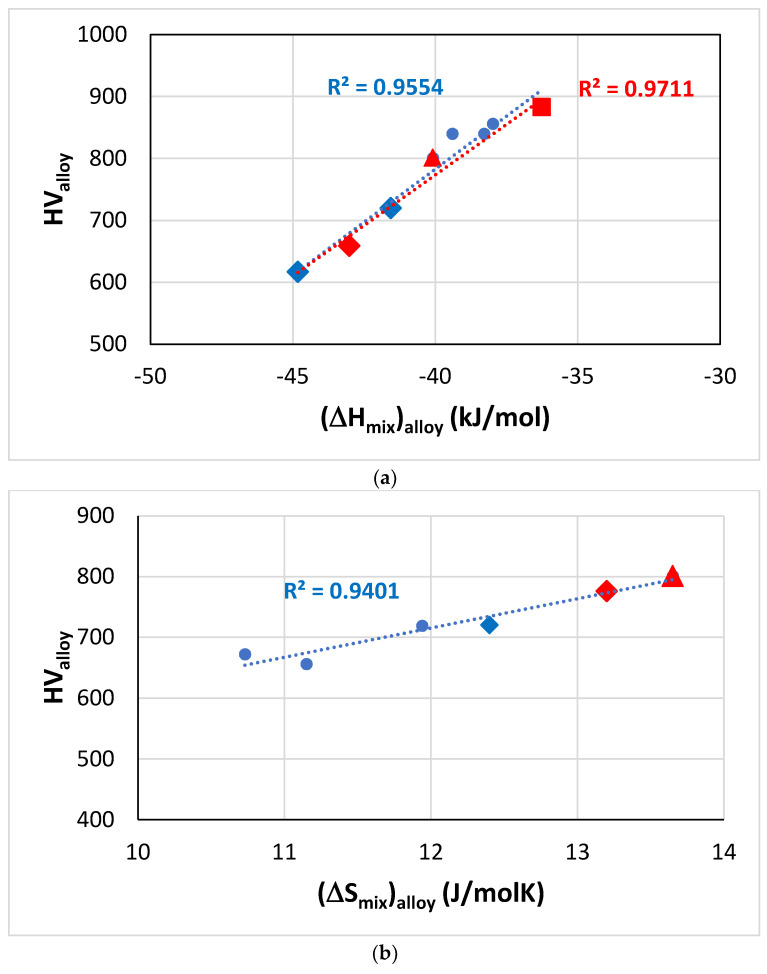

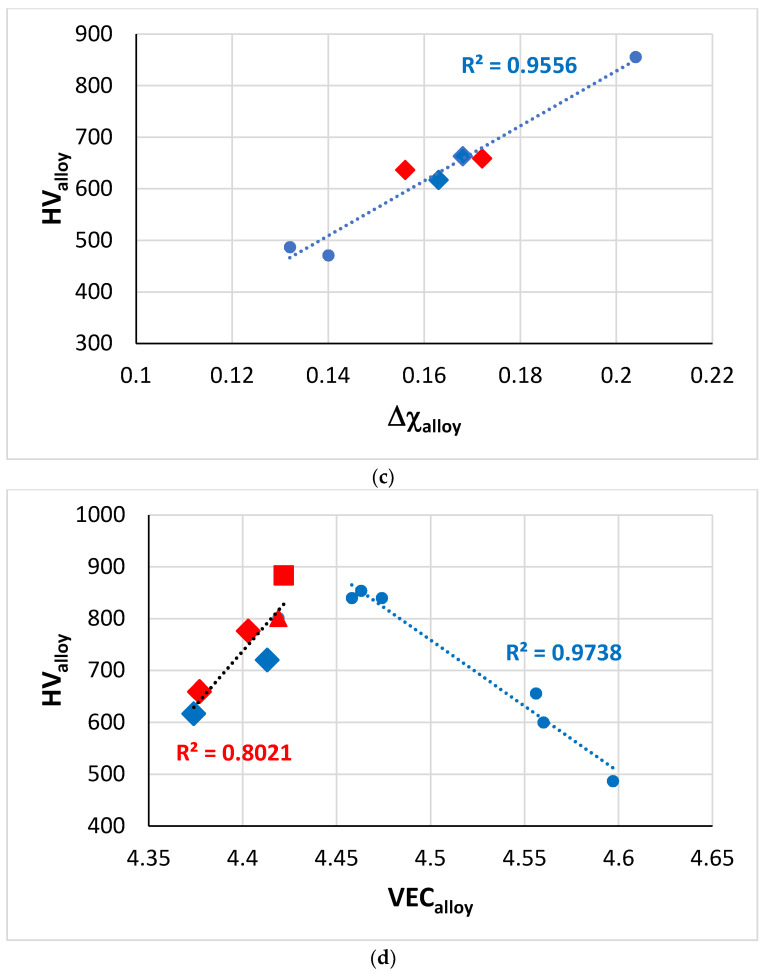
Vickers hardness of RM(Nb)ICs based on the alloy KZ5, some of which are also RCCAs (red data). Diamonds for alloys with B addition, square for RCCA with Sn addition and triangle for RCCA with Ge addition. (**a**) HV_alloy_ versus (ΔH_mix_)_alloy_, the linear fit of all data gives R^2^ = 0.9554, and only of the RCCAs gives R^2^ = 0.9711. Alloying elements Al, B, Cr, Ge, Hf, Mo, Nb, Si, Sn, Ti, alloys JN1, JN4, ZF6, ZF9, EZ8, KZ5 + B, KZ5 + Hf + B, (**b**) HV_alloy_ versus (ΔS_mix_)_alloy_, alloying elements Al, B, Cr, Ge, Hf, Mo, Nb, Si, Ti, W, alloys KZ5, ZF9, KZ5 + B, KZ5 + Hf + B, YG10, YG11, (**c**) HV_alloy_ versus Δχ_alloy_, alloying elements Al, B, Cr, Hf, Mo, Nb, Si, Sn, Ti, Ta, alloys KZ6, JG3, JN4, KZ5 + Mo + B, KZ5 + B, KZ6 + B, KZ5 + Hf + B, (**d**) HV_alloy_ versus VEC_alloy_, alloying elements Al, B, Cr, Ge, Hf, Mo, Nb, Si, Sn, Ti, Ta, alloys KZ5 + B, KZ5 + Hf + B, EZ8, ZF9, KZ5, JN1, ZF6, KZ6. Note that all the data in (**d**) can be fitted to a parabolic function with R^2^ = 0.9101 and maximum near the HV and VEC values of the alloy ZF6 (Nb-24Ti-18Si-5Al-5Cr-5Ge [34]). For nominal alloy compositions, see Appendix A Table A1.

The Vickers hardness of RM(Nb)ICs, some of which are also RCCAs, increases with increasing (ΔH_mix_)_alloy_, (ΔS_mix_)_alloy_ or Δχ_alloy_ (Figure 11a,c). Only the parameter VEC_alloy_ can separate the hardness of RM(Nb)ICs-RCCAs with B, Ge or Sn addition, which increases with VEC_alloy_, from the hardness of RM(Nb)ICs that are not RCCAs, which decreases with VEC_alloy_ (Figure 11d). The latter trend was also exhibited in the room temperature strength (calculated from hardness) versus the VEC_alloy_ plot discussed in [3], where it should be noted that the data includes Ti-free RM(Nb)ICs.

**Figure 12:**
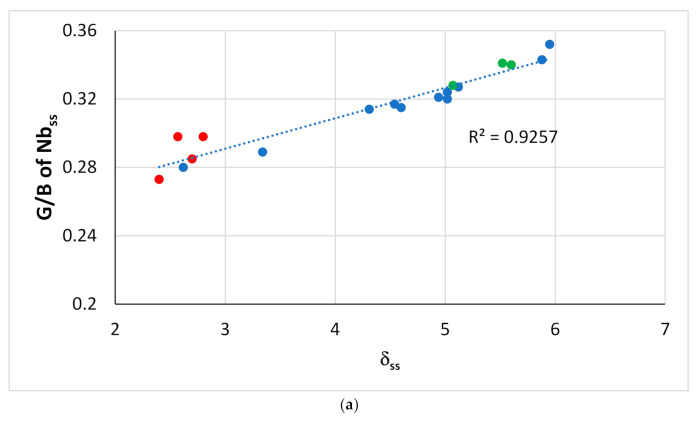

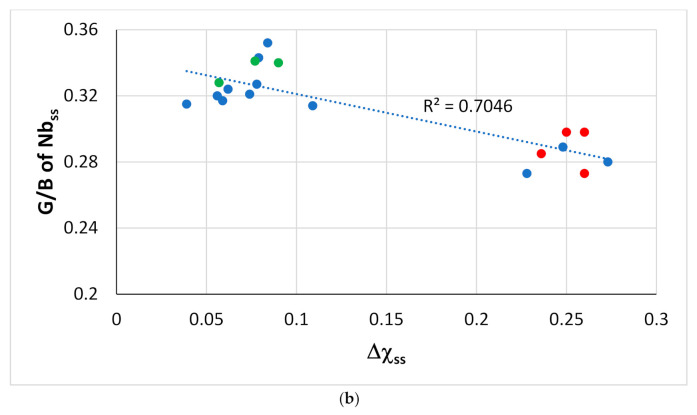
Pugh’s ratio G/B of Nb_ss_ in boron-free RM(Nb)ICs with/without Ti addition versus (**a**) δ_ss_ and (**b**) Δχ_ss_. Data for Si-free Nb_ss_ and Ti-rich Nb_ss_ is shown in red and green, respectively. All data R^2^ = 0.9257 and R^2^ = 0.7046, in (**a**,**b**), respectively. The G/B ratio was calculated using the rule of mixtures, data for G and B for the elements and the actual composition of solid solutions. Alloying elements Al, Cr, Ge, Hf, Mo, Nb, Si, Ta, Ti, W.

**Figure 13:**
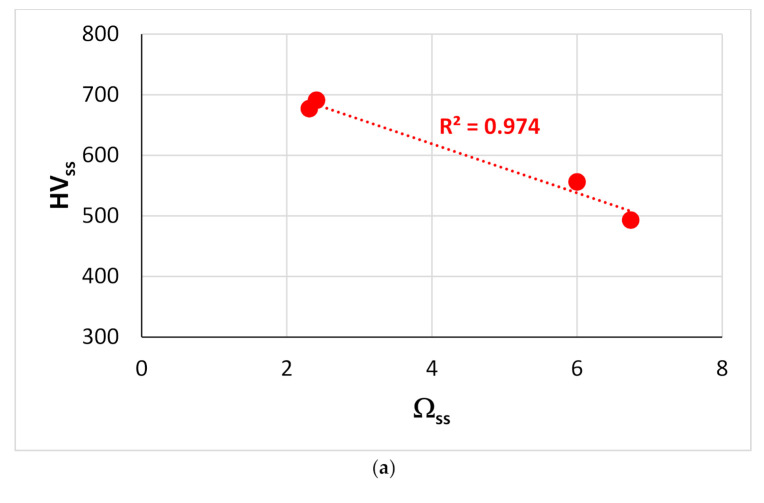

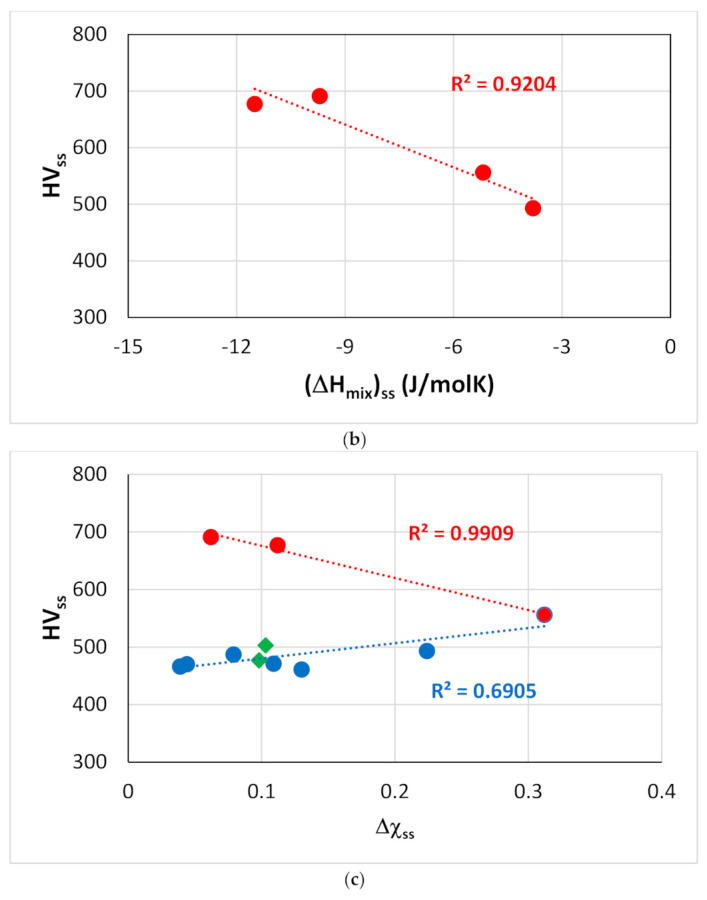
Vickers hardness of solid solutions in RM(Nb)ICs versus the parameters (**a**) Ω_ss_, (**b**) (ΔH_mix_)_ss_ and (**c**) Δχ_ss_. The red data in (**a**–**c**) is for alloying elements Al, Cr, Ge, Hf, Mo, Nb, Si, Sn, Ti or W. In (**c**), the data for which liner fit gives R^2^ = 0.6905 (blue line) is for alloying elements Al, B, Cr, Hf, Mo, Nb, Si, Sn, Ta, Ti or W. In (**c**) the data for the Nb_ss_ that belongs in RM(Nb)ICs that are also RCCAs is shown by green diamonds and is for alloys with B addition.

Figure 12 shows that the G/B ratio (Pugh’s ratio) of the bcc solid solution formed in RM(Nb)ICs without B addition, respectively, increases and decreases with the parameters δ_ss_ and Δχ_ss_ of the solid solution (the ratio G/B of the Nb_ss_ also decreases with VEC_ss_, figure not shown). Pugh [35] predicted ductile behavior for G/B < 0.5. Figure 13 shows the Vickers hardness of solid solutions (HV_ss_) formed in RM(Nb)ICs versus the parameters (a) Ω_ss_, (b) (ΔH_mix_)_ss_ and (c) Δχ_ss_. The HV_ss_ decreases with Ω_ss_, (ΔH_mix_)_ss_ or Δχ_ss_ for the solid solutions with Al, Cr, Ge, Hf, Mo, Nb, Si, Sn, Ti or W and increases with Δχ_ss_ for solid solutions that contain B and Ta, but not Ge. None of the solid solutions in this figure is a HEA/RHEA/RCCA, but the two solid solutions that belong in RM(Nb)ICs that are also RCCAs were formed in alloys with B addition. Note that the hardness HV_ss_ of Nb_ss_ in B-free as-cast RM(Nb)ICs increases with δ_ss_ [3].

**Figure 14:**
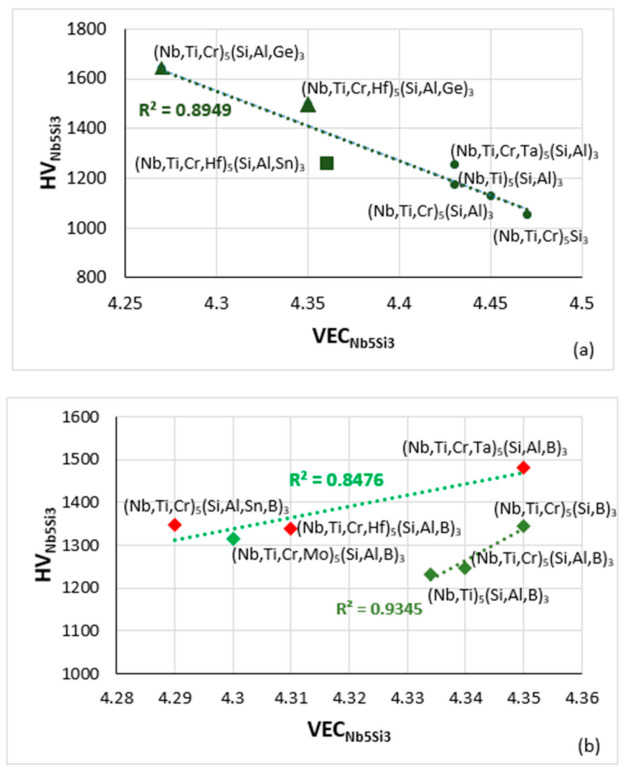
Vickers hardness of alloyed Nb_5_Si_3_ versus VEC. (**a**) Boron-free RM(Nb)ICs, triangles and squares with Ge or Sn addition, respectively, (**b**) boron-containing RM(Nb)ICs (diamonds), of which those that also are RHEAS and RCCAs are shown in red.

**Figure 15:**
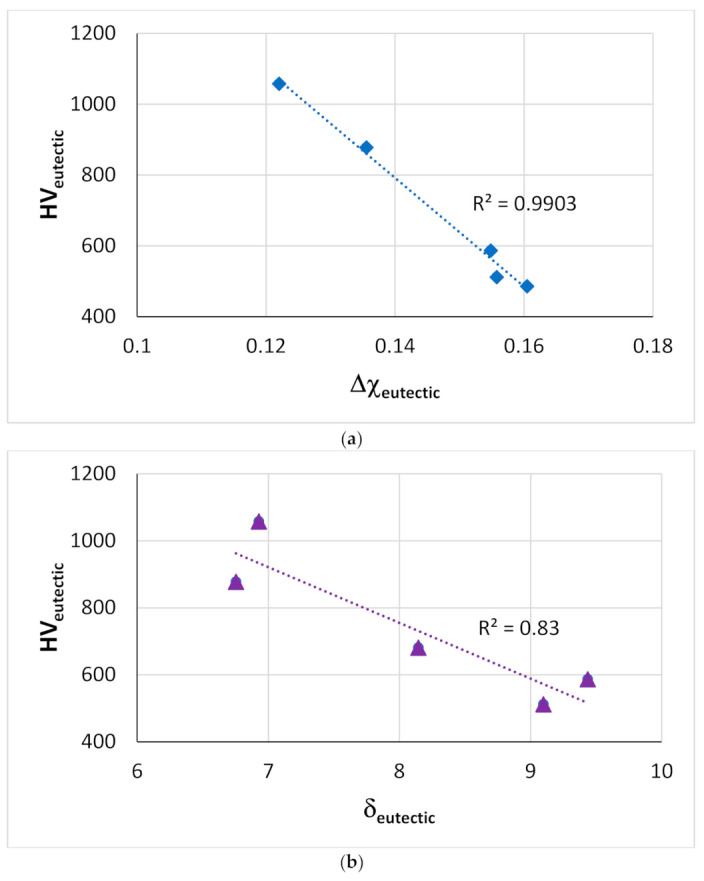
Vickers hardness of eutectics with Nb_ss_ and Nb_5_Si_3_ in Ti-free RM(Nb)ICs with alloying elements (**a**) Al, Cr, Ge, Hf, Nb, Si, Sn and (**b**) Cr, Ge, Hf, Nb, Si, Sn.

The effect of alloying additions on the hardness of Nb_5_Si_3_ was discussed in [9], where it was shown (i) that among the elements that substitute Si, the addition of Ge has the strongest effect regarding the increase of hardness, whereas Al, B and Sn decrease the hardness, and (ii) that among the elements that substitute Nb the addition of Cr, Hf and Ti has the opposite effect reducing the hardness compared with the binary Nb_5_Si_3_ [36,37]. Figure 14 shows that the Vickers hardness of alloyed Nb_5_Si_3_ decreases with VEC_Nb5Si3_ for RM(Nb)ICs without B addition that is not RCCAs (Figure 14a), whereas the HV_Nb5Si3_ increases with VEC_Nb5Si3_ for RM(Nb)ICs with B addition (Figure 14b). Note that the data in Figure 14b (a) does not include alloys with simultaneous addition of B and Ge, but (b) includes data for Nb_5_Si_3_ in RM(Nb)ICs that are also RCCAs (red diamonds). In RM(Nb)ICs, the Vickers hardness of eutectics with Nb_ss_ and Nb_5_Si_3_ increases with VEC_eutectic_ [11] and decreases with increasing Δχ_eutectic_ or δ_eutectic_ in Ti-free RM(Nb)ICs, respectively with Al, Cr, Ge, Hf, Nb, Si, Sn and Cr, Ge, Hf, Nb, Si, Sn alloying elements (Figure 15).

In RM(Nb)ICs, the Vickers hardness of alloyed A15-Nb_3_X (X = Al, Ge, Si or Sn) increases with increasing Δχ_A15_ or VEC_A15_ [10]. The increase of the creep rate of alloyed Nb_5_Si_3_ compared with the binary silicide [1] is linked with the shift of the position of Nb_5_Si_3_ in parameter maps (Figure 6, and [9]), namely with changes in the parameters Δχ_Nb5Si3_ and VEC_Nb5Si3_. Furthermore, there are relationships between the creep rate of RM(Nb)ICs and the parameters δ_alloy_, Δχ_alloy_ or VEC_alloy_ [1]. Unfortunately, there is no data about the creep of RHEAs and RCCAs and about the properties of the M_5_Si_3_ and Laves phase(s) that are observed in these UHTMs. No RHEAs and RCCAs with A15-Nb_3_X compounds have been studied to date outside our research group [19,25,26].

**Figure 16:**
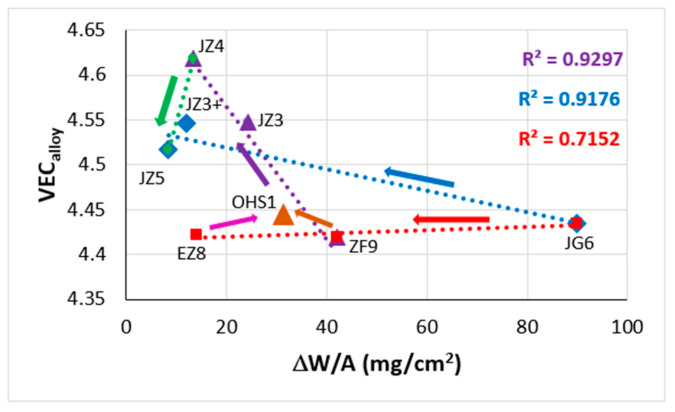
Data for the weight change in isothermal oxidation in air at 1200 °C of RM(Nb)ICs that are also RCCAs (data from Table 4 in [19]). Red squares for alloys where scale spalls off. Note that only the alloys EZ8, OHS1, JG6 and ZF9 are based on the alloy KZ5. For the nominal compositions of the alloys, see Appendix A Table A1.

Weight changes of alloys in isothermal oxidation are also linked with changes of the aforementioned parameters [1,19,26,27,38]. Figure 16 shows the weight change in isothermal oxidation at 1200 °C in the air of RM(Nb)ICs that are also RCCAs (data from Table 4 in [19]). Note (a) that the scale formed on the RM(Nb)ICs-RCCAs EZ8, JG6, ZF9 spalled off [19] (for the nominal composition of alloys see Table A1) and (b) that B-containing RM(Nb)ICs and RM(Nb)ICs-RCCAs do not experience pest oxidation at 800 °C and scale spallation at 1200 °C [3]. The arrows in Figure 16 indicate “direction of change” with alloying. The red arrow shows the effect on ΔW/A of removing Mo from its synergy with Al, Cr, Hf and Sn in the alloy JG6 [39] and having Ge in synergy with Al, Cr and Hf in ZF9 [34] or Sn in synergy with Al, Cr and Hf in EZ8. The brown and pink arrows show the shift towards the Hf-free alloy OHS1 [26], where Ge and Sn were in synergy with Al and Cr. The blue arrow shows the change owing to having Ge and Sn in synergy with Al, Cr, Hf and Ta (JZ3+ [25]) or Mo (JZ5 [19]). The purple arrow shows the effect of adding to OHS1 the elements Hf and Ta (JZ3 [25]) or Hf and Mo (JZ4 [19]), and the green arrow shows the effect of increasing the Ti concentration (JZ5). Note that the alloys JZ5 and JZ4 belong in the Nb-Mo-W-Ti-Cr-Hf-Al-Ge-Si-Sn system (see the second paragraph of Section 2 and Section 5).

The densities of RM(Nb)ICs, RM(Nb)ICs with B addition and RM(Nb)ICs-RCCAs with B addition, respectively are in the ranges 6.27 < ρ < 8.67 g/cm^3^, 6.41 < ρ < 6.87 g/cm^3^ and 6.46 < ρ < 6.87 g/cm^3^ [3] whereas the densities of RM(Nb)ICs-RCCAs with Ge or Sn or Ge + Sn and with/without Hf addition are in the range 6.78 < ρ < 7.94 g/cm^3^ [19]. The densities of the RCCAs reviewed in [13] were in the range 5.6 < ρ < 13.8 g/cm^3^ with those with Al, or Cr with/without Si, Ti, V or Zr addition having ρ < 9.08 g/cm^3^ whereas the RCCAs with high strength at T ≥ 1200 °C have ρ > 10 g/cm^3^. The room temperature specific strength of RM(Nb)ICs with B addition and RM(Nb)ICs-RCCAs with B addition calculated from hardness, respectively, is in the ranges 315.8 < σ^HV^/ρ < 376.5 MPa cm^3^ g^–1^ and 340.2 < σ^HV^/ρ < 383.6 MPa cm^3^ g^–1^ [3], while that of the alloys JZ4 and JZ5 with Ge + Sn addition, respectively was 387 and 396 MPa cm^3^ g^–1^ [19], higher than the specific strength of multiphase RHEAs and RCCAs that is less than about 308 MPa cm^3^ g^–1^ [13]. In other words, RM(Nb)ICs-RCCAs with the addition of Be, Ge, or Sn have superior room temperature strength and specific strength and do not experience pest oxidation and scale spallation compared with RHEAs and RCCAs without these solutes.

## 4. Alloying Behavior of RM(Nb)ICs, RHEAs and RCCAs, Relationships Between Alloys and Their Phases

**Figure 17:**
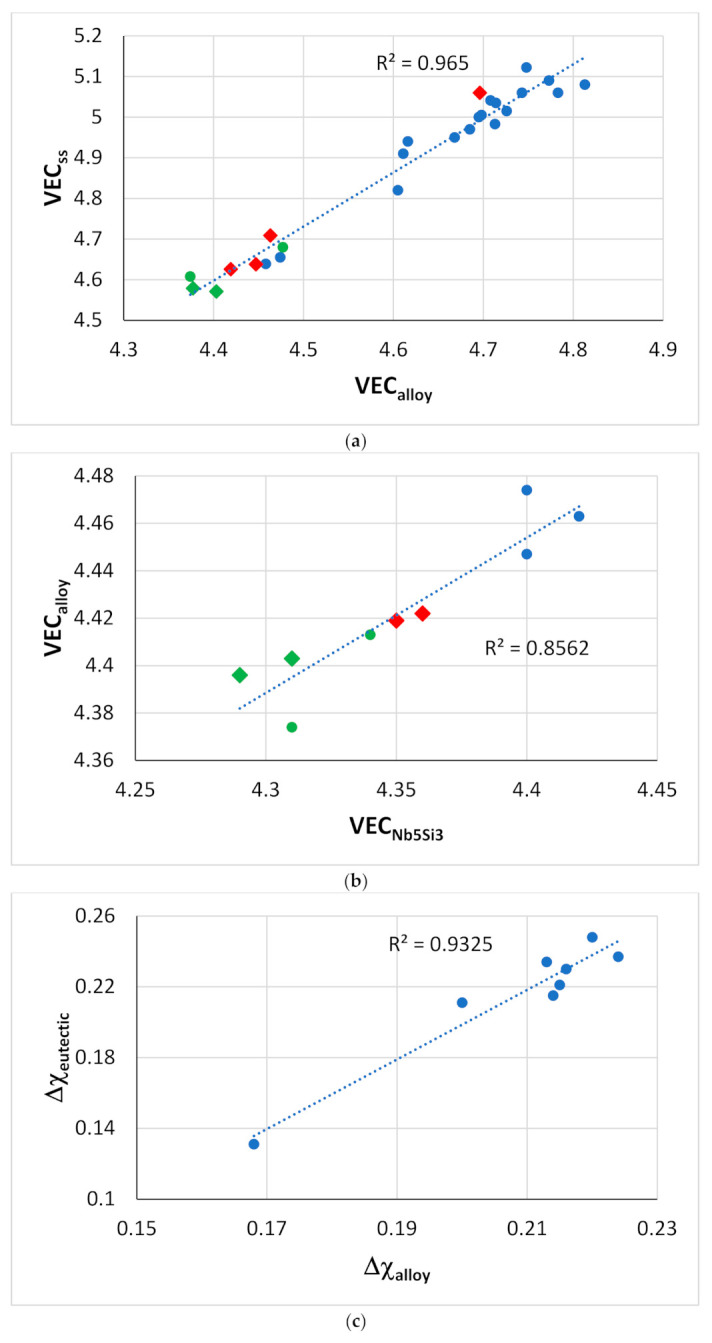
Relationships between parameters of alloys and phases. (**a**) VEC_ss_ versus VEC_alloy_ [1], diamonds for RM(Nb)ICs that are also RCCAs, green data for alloys with B, alloying elements Al, B, Cr, Ge, Hf, Mo, Nb, Si, Sn, Ti or W, (**b**) VEC_alloy_ versus VEC_Nb5Si3_, circles RM(Nb)ICs, green data alloys with B, diamonds for RM(Nb)ICs that are also RCCAs, alloying elements Al, B, Cr, Ge, Hf, Nb, Si, Sn or Ti, (**c**) Δχ_eutectic_ versus Δχ_alloy_ for eutectics with Nb_ss_ and Nb_5_Si_3_, alloying elements Al, Cr, Ge, Hf, Mo, Nb, Si, Sn, Ti or W.

**Figure 18:**
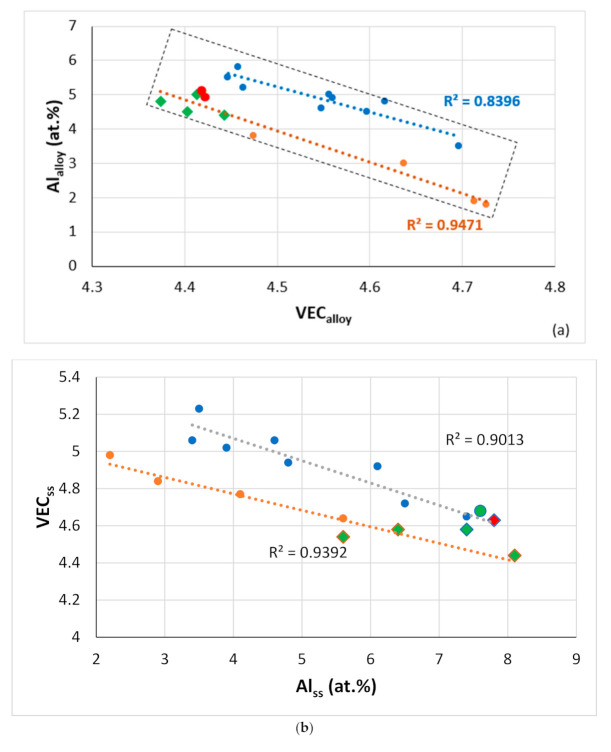

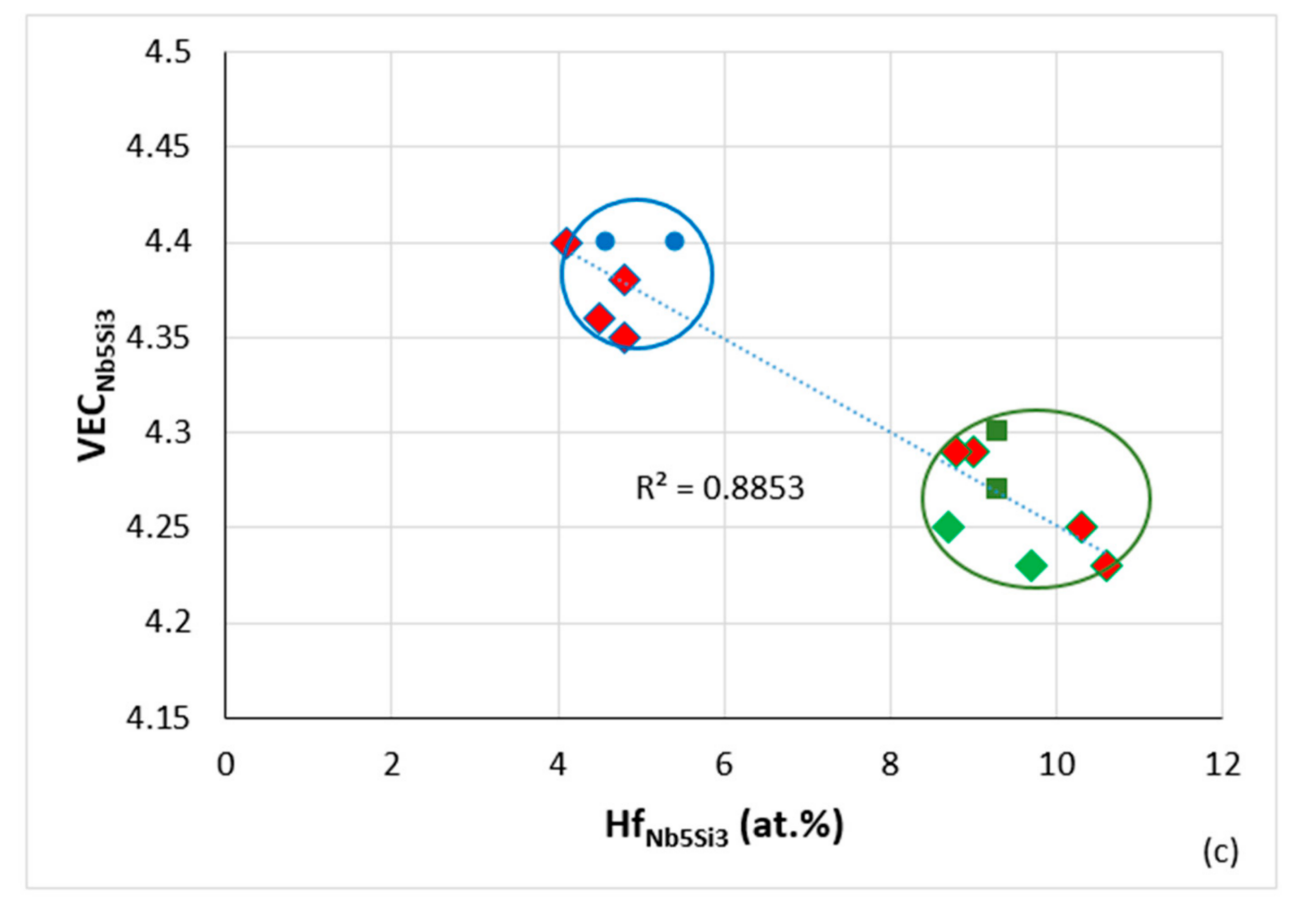
Relationships between alloy or phase parameters and solute concentrations in RM(Nb)IC and RCCAs alloys with Ti/Si < 1 and Ti/Si > 1 [3] and their phases. (**a**) Al_alloy_ versus VEC_alloy_, orange line (R^2^ = 0.9471) with green diamonds for alloys with B, green and red data points for RM(Nb)ICs that are also RCCAs, alloying elements Al, B, Cr, Ge, Hf, Mo, Nb, Si, Sn, Ta, Ti or W, blue line (R^2^ = 0.8396) for alloying elements Al, Cr, Ge, Hf, Mo, Nb, Si, Sn, Ta, Ti or W, (**b**) VEC_ss_ versus Al_ss_, orange line (R^2^ = 0.9392) for alloying elements Al, B, Cr, Ge, Hf, Mo, Nb, Si, Sn, Ti, V or W, diamonds for B-containing RM(Nb)ICs that are also RCCAs, blue line (R^2^ = 0.9013) for alloying elements Al, B, Cr, Ge, Hf, Mo, Nb, Si, Sn, Ta, Ti, or W, circles for RM(Nb)ICs, diamonds for RM(Nb)ICs that are also RCCAs, green color for B-containing alloys, (**c**) VEC_Nb5Si3_ versus the concentration of Hf in Nb_5_Si_3_, green circle for Ti rich Nb_5_Si_3_, blue circle for normal Nb_5_Si_3_, for all data R^2^ = 0.8853, diamonds for RM(Nb)ICs that are also RCCAs, green diamonds for B-containing alloys, alloying elements Al, B, Cr, Ge, Hf, Nb, Si, Sn, Ti, linear fit for data for RM(NB)ICs that are also RCCAS has R^2^ = 0.9209. For the rectangular area in (**a**), see text.

In view of the relationships between properties of the alloys and the aforementioned parameters (Figure 11, Figure 12, Figure 13, Figure 14, Figure 15 and Figure 16), one would expect that the latter can also link alloys and their phases. Indeed, this is the case. Figure 17 shows (i) that the parameter VEC can link alloys and their Nb_ss_ and Nb_5_Si_3_ and (ii) that the parameter Δχ links alloys and their eutectics with Nb_ss_ and Nb_5_Si_3_ (relationships also exist with the parameter δ, not shown in this paper). As the Al content in the alloy increases, the VEC_alloy_ decreases (Figure 18a), and the VEC_ss_ decreases (Figure 17a) as the Al of the Nb_ss_ increases (Figure 18b). Note that in Figure 18a, the rectangle defines the area for alloys with different Ti/Si ratios [3] and alloying element additions. In this area, the B-containing RM(Nb)ICs and RM(Nb)ICs-RCCAs are close to the lower bound, whereas RM(Nb)ICs are closer to the upper bound.

Both Hf and Ti partition to the Nb_5_Si_3_, where they substitute Nb [9]. Ti-rich Nb_5_Si_3_ is also rich in Hf compared with the “normal” Nb_5_Si_3_ [9]. The partitioning of Hf with Ti in Ti-rich Nb_5_Si_3_ contributes to the decrease of VEC_alloy_ (Figure 17b) owing to the decrease of the VEC_Nb5Si3_ (Figure 18c). Ti (and Hf) rich Nb_5_Si_3_ can form in as-cast RM(Nb)ICs and, unlike Ti-rich Nb_ss_, can be present after heat treatment. The partitioning of Ti and Hf (and other solute additions) in Nb_5_Si_3_ affects its hardness (Figure 14). Changes in VEC_Nb5Si3_ are also linked with changes in Δχ_Nb5Si3_ (Figure 6), which together with changes in Δχ_ss_ [1,3,7], can affect the properties of eutectics with Nb_ss_ and Nb_5_Si_3_. For the latter, the parameter Δχ_eutectic_ can increase or decrease with <Si> = Al + Ge + Si + Sn depending on the alloying additions [11]. For the alloying additions given in the caption of Figure 17c, the increase of Δχ_eutectic_ with Δχ_alloy_ is linked with a decrease of <Si> [11] and thus with an increase of VEC_eutectic_ and HV_eutectic_ [11].

**Figure 19:**
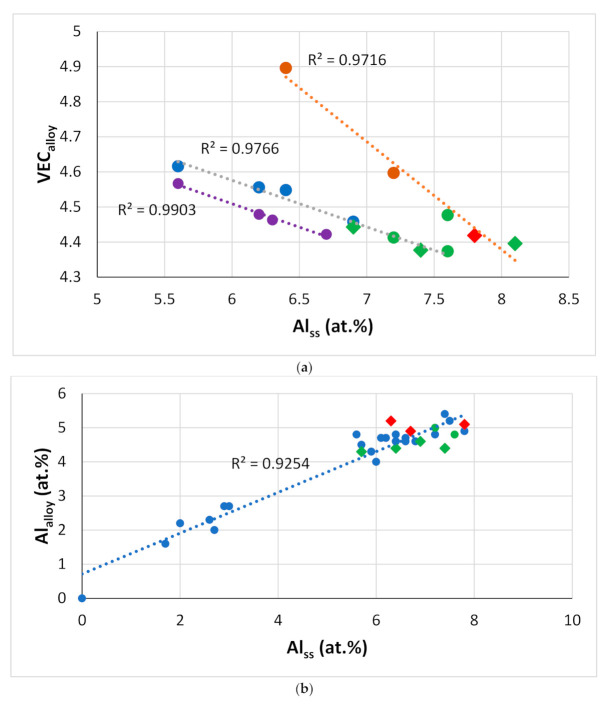

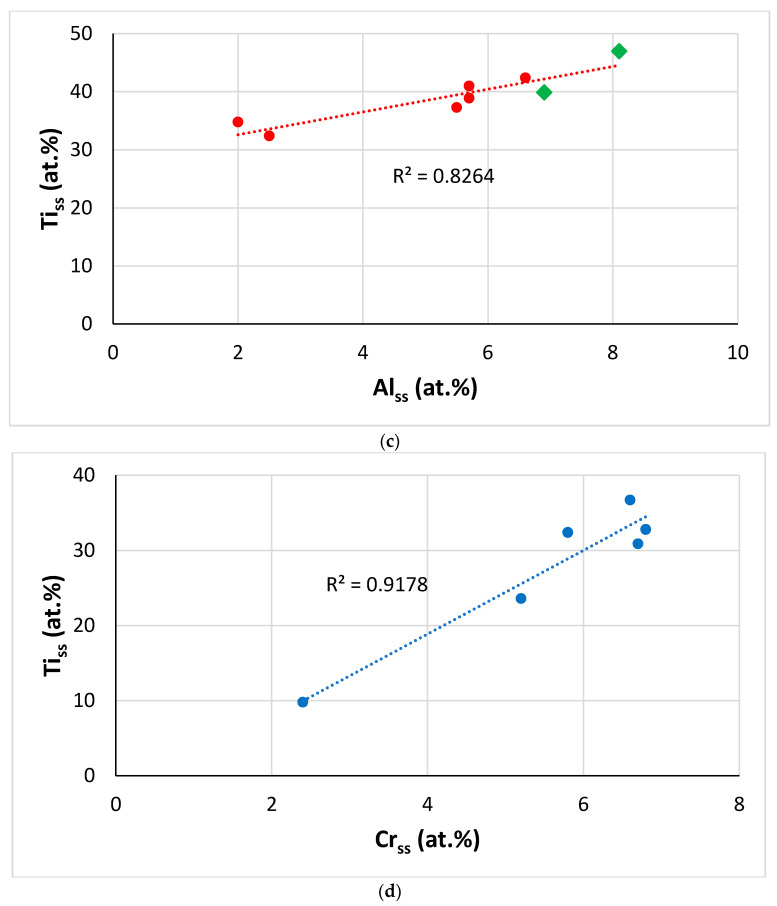
Data for alloys and bcc solid solutions for RM(Nb)ICs, some of which are also RCCAs (**a**) VEC_alloy_ versus Al_ss_, orange line (R^2^ = 0.9716) with alloying elements Al, B, Cr, Hf, Mo, Nb, Si, Sn, Ta, Ti, or W, blue line (R^2^ = 0.9766) with alloying elements Al, B, Cr, Ge, Hf, Mo, Nb, Si, Ta, Ti or W, purple line (R^2^ = 0.9903) with alloying elements Al, Cr, Ge, Hf, Nb, Si, Sn, Ti or V, (**b**) Al_alloy_ versus Al_ss_, alloying elements Al, B, Cr, Ge, Hf, Mo, Nb, Si, Sn, Ta, Ti or W, (**c**) Ti_ss_ versus Al_ss_ with alloying elements B, Cr, Fe, Hf, Sn or Ta, (**d**) Ti_ss_ versus Cr_ss_ with alloying elements Al, Fe, Hf, Sn, W or V. In (**a**–**c**) diamonds for RM(Nb)ICs that are also RCCAs, and green colour for alloys with B.

Given that the aforementioned parameters connect alloys and their phases (Figure 11, Figure 12, Figure 13, Figure 14, Figure 15, Figure 16, Figure 17 and Figure 18), one would expect relationships that link solute additions in alloys and their phases. In actual fact, this is the case. The concentrations of Ti, Al or W in the bcc solid solution(s) in RM(Nb)ICs increase with the Ti, Al or W concentrations in the alloy (e.g., Figure 19b and [1]). As Ti partitions to the solid solution, it “pulls” with it Al and Cr (Figure 19c,d and [40]) and “pushes away” W [19,25,41]. The changes of the solute concentrations in the alloy and its solid solution(s) are associated with changes in parameters (e.g., Figure 17a, Figure 18a,b and Figure 19a) and properties of the solid solution (e.g., Figure 12 and Figure 13).

**Figure 20:**
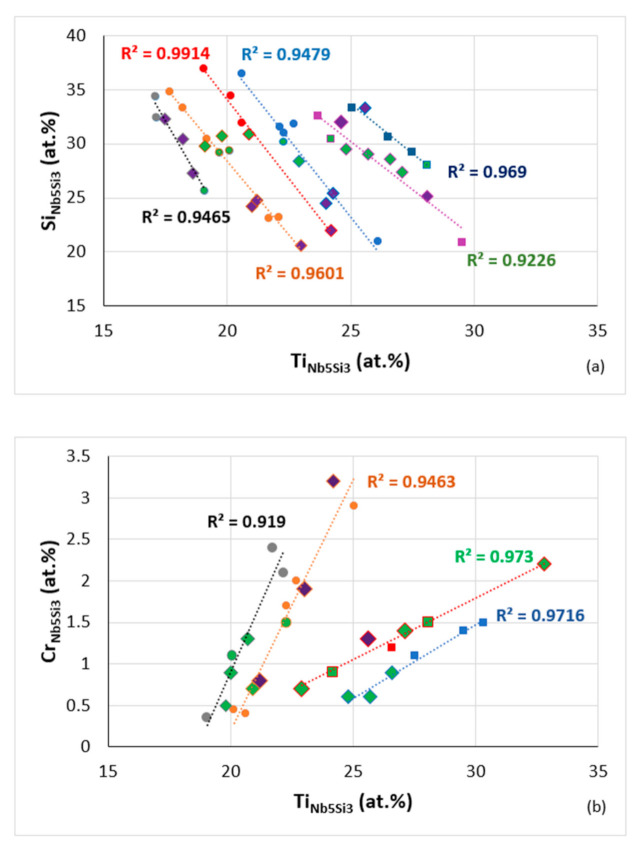

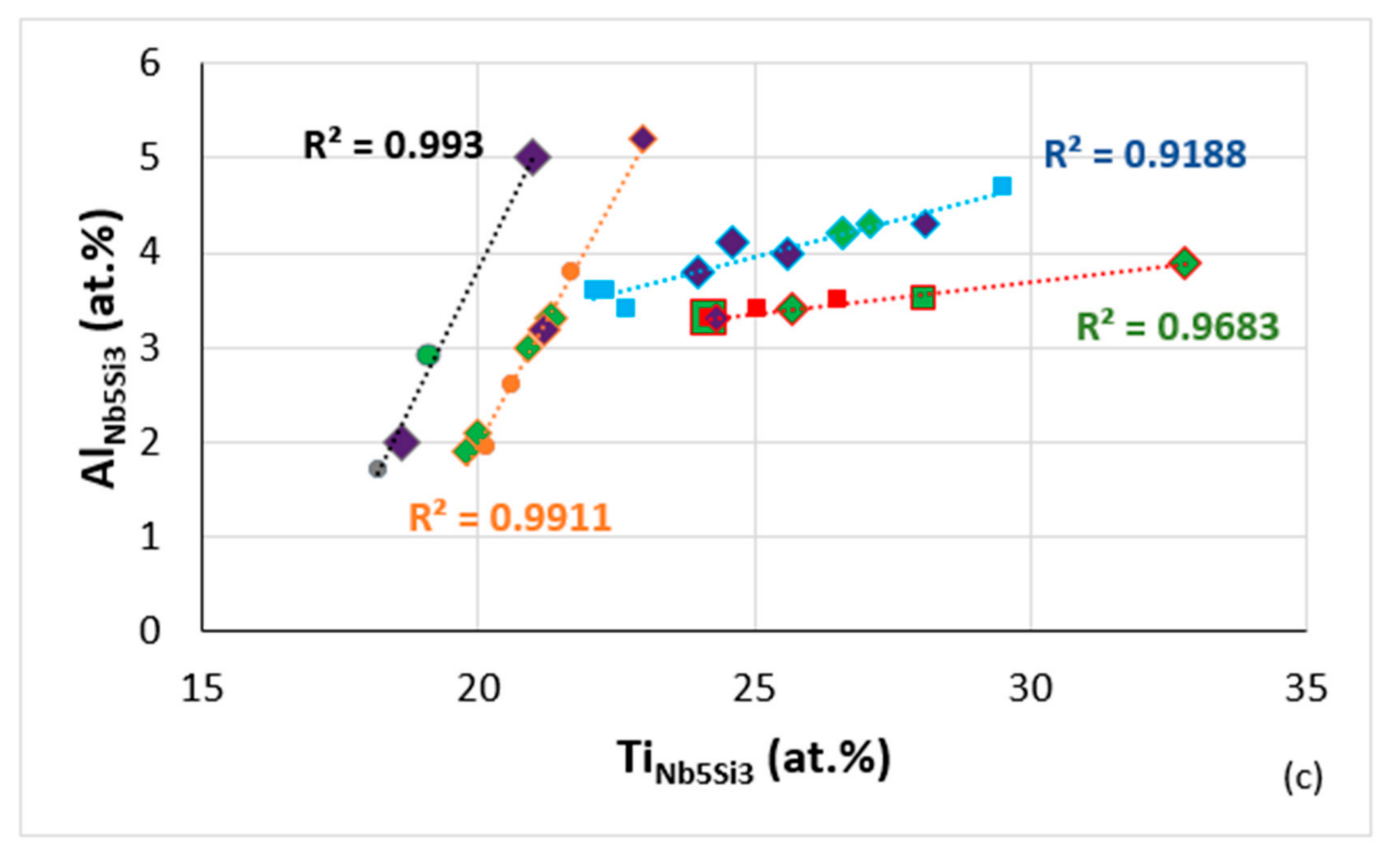
Data for solute elements in Nb_5_Si_3_ in RM(Nb)ICs some of which are also refractory complex concentrated alloys (RCCAs) (diamonds), silicides with B addition shown in green colour, purple diamonds RCCAs with Ge or Sn or Ge + Sn, (**a**) Si versus Ti, R^2^ = 0.9465 alloying elements Al, B, Cr, Ge, Hf, Mo, Nb, Si, Sn or Ti, R^2^ = 0.9601 alloying elements Al, B, Cr, Ge, Hf, Mo, Nb, Si, Sn, Ta or Ti, R^2^ = 0.9914 alloying elements Al, B, Cr, Ge, Nb, Si, Sn, Ta or Ti, R^2^ = 0.9479 alloying elements Al, B, Cr, Ge, Hf, Mo, Nb, Si, Sn, Ta or Ti, R^2^ = 0.9226 for Ti rich Nb_5_Si_3_ alloying elements Al, B, Cr, Ge, Hf, Mo, Nb, Si, Sn, Ta or Ti, R^2^ = 0.969 for Ti rich Nb_5_Si_3_ alloying elements Al, B, Cr, Hf, Mo, Nb, Si, Sn, Ta or Ti, (**b**) Cr versus Ti, R^2^ = 0.919, alloying elements Al, B, Cr, Ge, Hf, Mo, Nb, Si, Sn, Ta or Ti, R^2^ = 0.9463, alloying elements Al, B, Cr, Ge, Hf, Mo, Nb, Si, Sn, Ta or Ti, R^2^ = 0.973, Ti rich Nb_5_Si_3_, alloying elements Al, B, Cr, Hf, Mo, Nb, Si, Sn, Ta or Ti, R^2^ = 0.9716, Ti rich Nb_5_Si_3_, alloying elements Al, B, Cr, Ge, Hf, Mo, Nb, Si, Sn, Ta or Ti, (**c**) Al versus Ti, R^2^ = 0.993, alloying elements Al, B, Cr, Ge, Hf, Mo, Nb, Si, Sn or Ti, R^2^ = 0.9911, alloying elements Al, B, Cr, Ge, Hf, Nb, Si, Sn, Ta or Ti, R^2^ = 0.9188, Ti rich Nb_5_Si_3_, alloying elements Al, B, Cr, Ge, Hf, Mo, Nb, Si, Sn, Ta or Ti, R^2^ = 0.9683, Ti rich Nb_5_Si_3_ alloying elements Al, B, Cr, Ge, Hf, Mo, Nb, Si, Ta or Ti.

In the same way, as there are relationships between parameters and solutes in the bcc solid solution in RM(Nb)ICs and RCCAs (Figure 19a), and between solutes in the solid solution (Figure 19b,d), there are relationships between solutes in Nb_5_Si_3_ and Δχ_Nb5Si3_ (Figure 2 in [9] for B, Ge and Sn), between Ti_Nb5Si3_ and B, Ge, Sn (Figure 1 in [9]), Al and Si in Nb_5_Si_3_ (Figure 8 in [42]), between Hf and Nb in Nb_5_Si_3_ (Figure 8 in [42]). Figure 20 shows data for Al, Cr and Si in Nb_5_Si_3_. Note that this figure includes data for RM(Nb)ICs that are also RCCAs. Furthermore, note that, as was the case for the bcc solid solution, the data for RCCAs follows the same trend as that for RM(Nb)ICs (remember that Ti “pulls” with it Hf in Nb_5_Si_3_). The changes of the solute concentrations in the alloy and its 5–3 silicide are associated with changes in parameters (e.g., Figure 17b and Figure 18c) and properties of the silicide (Figure 14).

**Figure 21:**
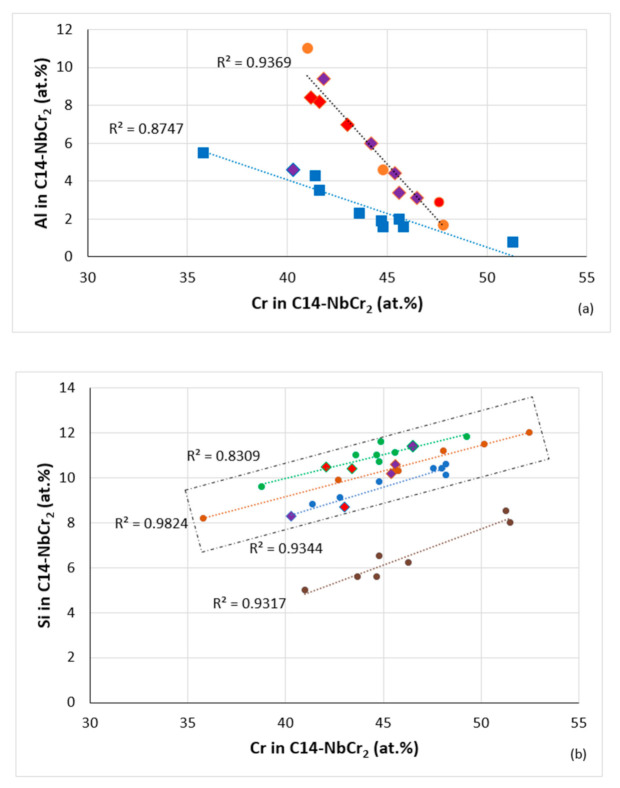
Data for Al or Cr in C14-NbCr_2_ Laves phase. For all data points, the Cr in Laves phase is substituted by Al, Ge, Si or Sn. Red data points for C14-NbCr_2_ Laves phase in oxidized alloys. (**a**) Al versus Cr in C14-NbCr_2_. Diamonds for RM(Nb)ICs that are also RCCAs. R^2^ = 0.9369, alloying elements Al, Cr, Ge, Hf, Mo, Nb, Si, Sn, Ta, Ti or W, R^2^ = 0.8747 alloying elements Al, Cr, Ge, Hf, Mo, Nb, Si, Sn, Ti or W. Red data points (R^2^ = 0.9991), (**b**) Si versus Cr in C14-NbCr_2_. The data with R^2^ = 0.9317 is for Ta-free alloys. R^2^ = 0.9317, alloying elements Al, Cr, Ge, Hf, Mo, Nb, Si, Sn, Ti or W, R^2^ = 0.9344, alloying elements Al, Cr, Ge, Hf, Mo, Nb, Si, Sn, Ta, Ti or W, R^2^ = 0.9824, alloying elements Al, Cr, Ge, Hf, Mo, Nb, Si, Sn, Ta, Ti or W, R^2^ = 0.8309, alloying elements Al, Cr, Ge, Hf, Mo, Nb, Si, Sn, Ta, Ti or W. For the rectangular area in (**b**), see text.

In addition, there exist relationships between solutes in the C14-NbCr_2_ Laves phase that can form in RM(Nb)ICs and RM(Nb)ICs-RCCAs. Figure 21 shows such relationships for Al and Si. Note that data for Laves phase in oxidized alloys are also included in this figure. As the Cr concentration in the Laves increases, the concentrations of Al and Si, respectively, decrease and increase. The rectangular area in Figure 21b indicates the range of Si and Cr concentrations in C14-NbCr_2_ Laves with upper (R^2^ = 0.8309) and lower (R^2^ = 0.9344) bands for RM(Nb)ICs and RM(Nb)ICs-RCCAs with RM = Mo,Nb,Ta,W, TM = Cr,Hf,Ti and SM-Met = Al,Ge,Si,Sn. The changes in solute concentrations in the Laves phase result in changes of its parameters VEC_C14-NbCr2_ and Δχ_C14-NbCr2_ and its properties [10].

## 5. The Alloy Design/Selection Methodology NICE

The above-mentioned interrelationships of alloys and their phases regarding (i) alloying behavior, (ii) properties and (iii) solute concentrations are captured in the alloy design methodology NICE, the development of which was based on data for RM(Nb)ICs and was expanded to include data for RM(Nb)ICs that are also HEAs, RHEAs and RCCAs. The aforementioned parameters of alloys and their phases were calculated for those alloys for which reliable chemical analysis data (EPMA and EDS with standards) was available [1,3]. The database is updated as new data becomes available. The framework of NICE was discussed first in [1] and then in [3]. In the ref. [3] the RM(Nb)ICs that were included in [1] and the RCCAs reviewed in [13] were compared.

The choice of method and procedure in NICE was not content-neutral but closely bound with the identification of difficulties with experimental, modeling and ab-initio research [1,3,13]. The different components in the conception support and reinforce one another. Basically, NICE is a “goal-driven” alloy design approach that leads to the selection of metallic UHTMs worthy of development owing to promising oxidation or creep properties. NICE does not consider toughness. In NICE, the design of an alloy combines constraint(s), say desirable alloying elements and/or phases or RM/TM, or SM/Met ratios in the alloy (e.g., see [19,20,21,25]), with a property goal, say a creep rate (έ) target or an oxidation weight change (ΔW/A) target, e.g., [19,25,43]. The alloy design starts with the calculation of the alloy composition. This is done using the relationship between, say, έ and Δχ_alloy_ to calculate the latter and then relationships of Δχ_alloy_ with the concentration of each element i (C_i_^alloy^) are used to calculate C_i_^alloy^ [1]. Clearly, elements with C_i_^alloy^ < 0 cannot be present in the alloy composition. The calculated alloy composition can be consistent with an RM(Nb)IC or an RM(Nb)IC-RHEA/RCCA.

The next step is to calculate first the parameters δ_alloy_, VEC_alloy_, ΔH_mix_^alloy^, ΔS_mix_^alloy^ and Ω_alloy_ for the alloy composition [1] and then alloy properties, for example, room temperature strength [3] and hardness (e.g., Figure 11). From the alloy composition, one can also calculate the macrosegregation of Si (MACSi) if the alloy were to be produced using liquid route processing [19,24,25,26,34,38,42,43,44]. Then one proceeds to calculate the composition of the bcc solid solution. First, the Δχ_Nbss_ is calculated from the relationship between Δχ_alloy_ and Δχ_Nbss_ [1]. The chemical composition of the solid solution is calculated from relationships between C_i_^Nbss^ and Δχ_Nbss_ (there are also relationships between C_i_^Nbss^ and δ_Nbss_ or VEC_Nbss_, e.g., Figure 18b). Properties of the solid solution, for example, hardness, are then calculated [3] (Figure 13). The solid solution is considered not to be stable in the alloy if 0.13 < Δχ_Nbss_ < 0.18 (Table 1, Figure 4). The type of solid solution, for example, Si-free Nb_ss_, is predicted from the δ_Nbss_ value. The latter is calculated from the δ_alloy_ versus δ_Nbss_ relationship [1]. Si-free Nb_ss_ is predicted to be stable in the alloy if δ_Nbss_ < 5 (Figure 3).

**Figure 22:**
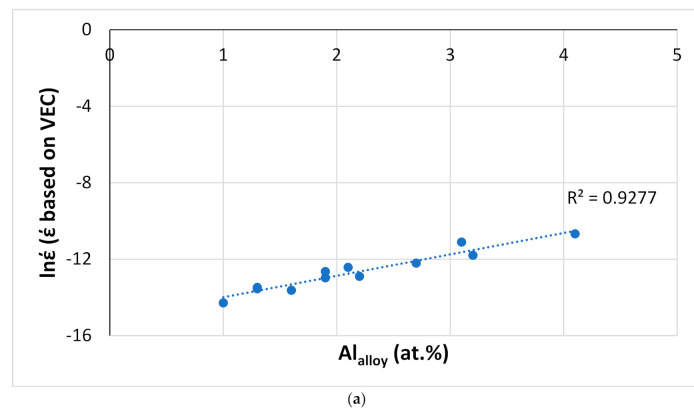

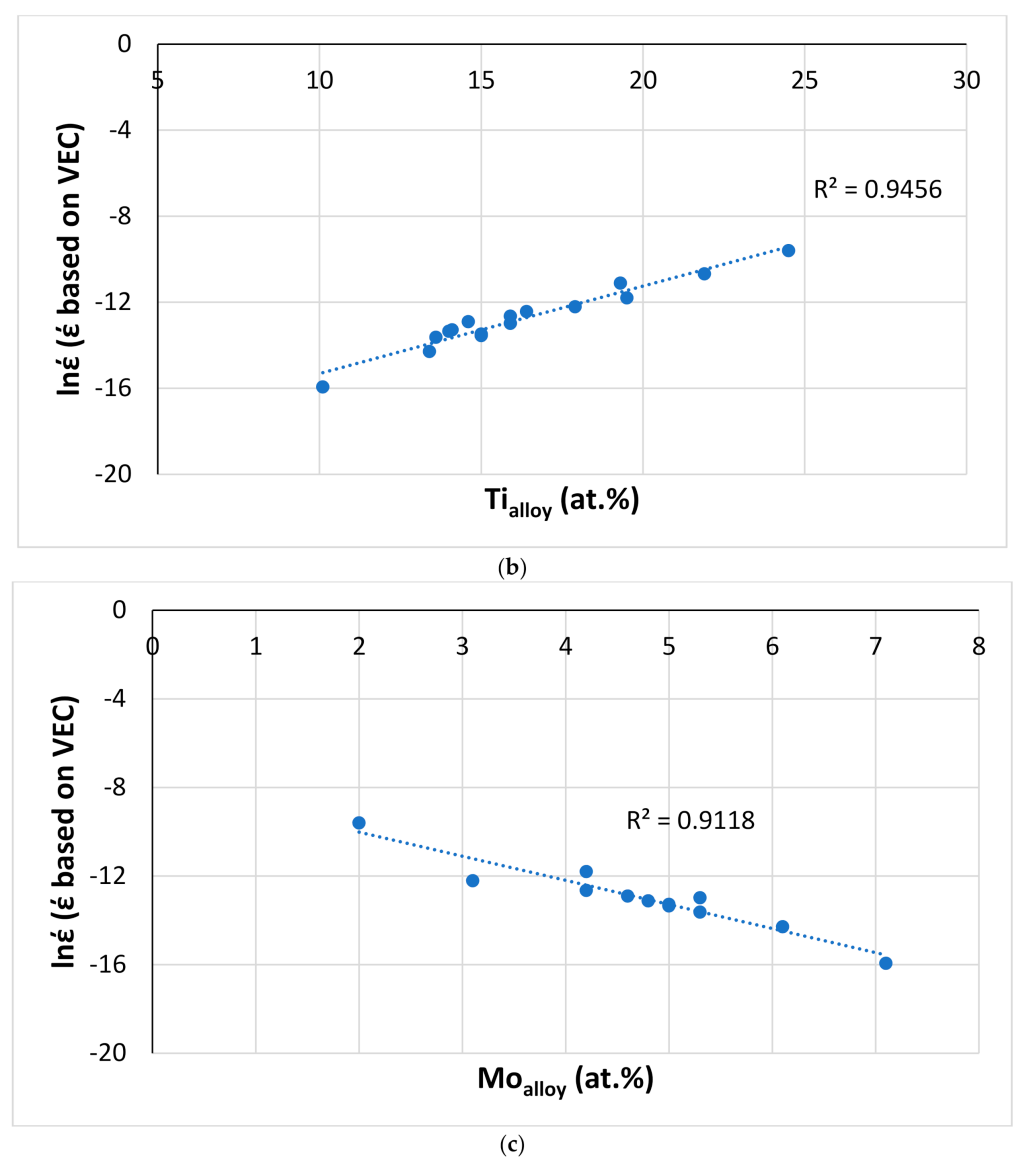

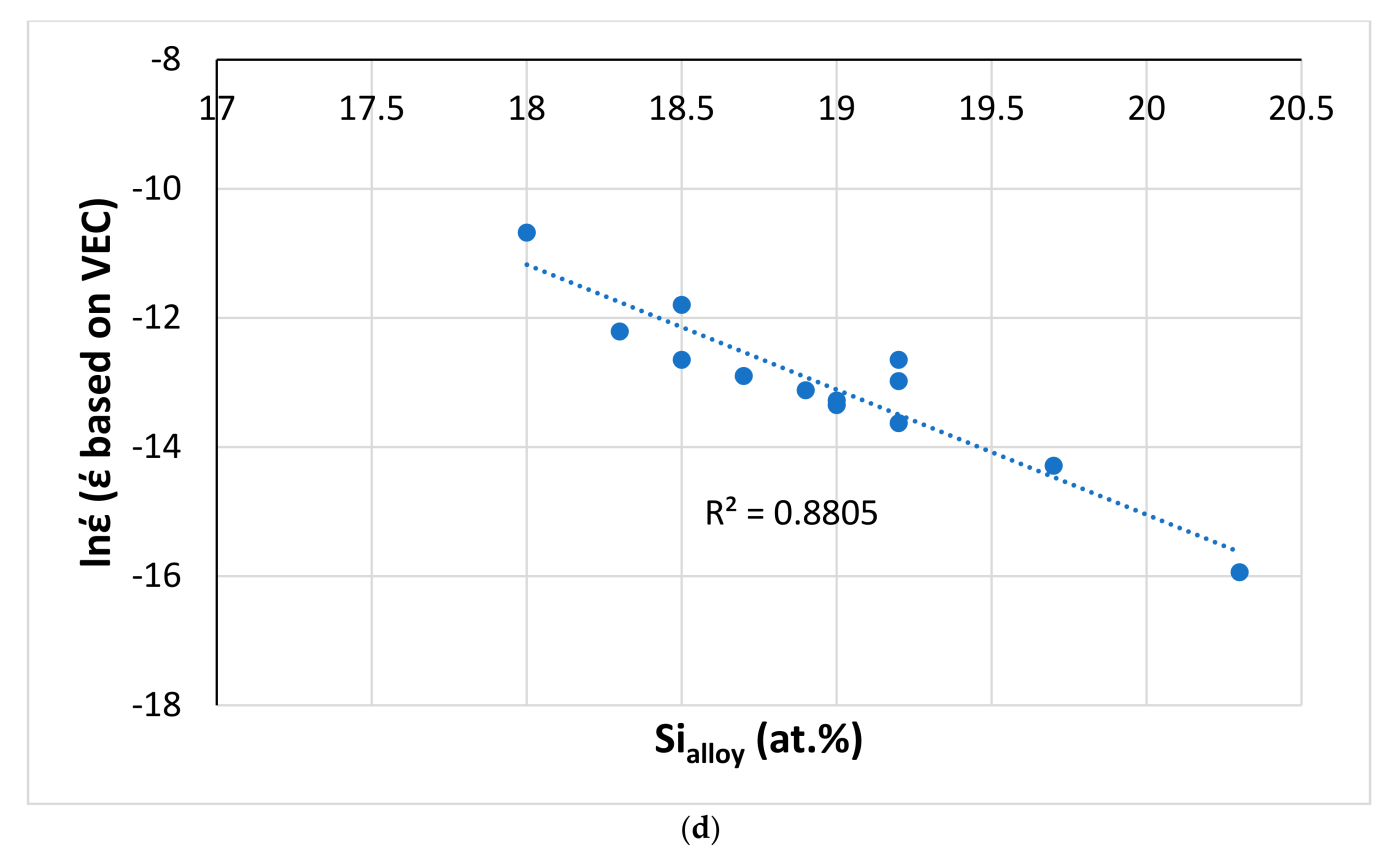
Calculated secondary creep rates-based on VEC for the creep goal conditions (T = 1200 °C, σ = 170 MPa) versus (**a**)Al, (**b**), Ti (**c**) Mo and (**d**) Si content in RM(Nb)ICs.

The chemical composition of intermetallic compounds is calculated following a similar approach. For example, for Nb_5_Si_3_, the Δχ_Nb5Si3_ is calculated from the relationship between Δχ_alloy_ and Δχ_Nb5Si3_ and then the concentration of each element i (C_i_^Nb5Si3^) is calculated from relationships between C_i_^Nb5Si3^ and Δχ_Nb5Si3_ [1]. The formation or not of a eutectic with Nb_ss_ and Nb_5_Si_3_ and whether the eutectic would be Ti-rich or Ti-poor [11] can be predicted using relationships between Δχ_alloy_ and Δχ_eutectic_ [1]. Similar to the alloy and phases, properties of the eutectic, for example hardness, can be calculated (e.g., see Figure 15). The vol. % of solid solution [19,25] and other properties also can be calculated. For example, mass changes (ΔW/A) after isothermal oxidation for 100 h at 800 or 1200 °C and steady-state creep rates έ for different temperatures and stresses are calculated using relationships between the alloy parameters and ΔW/A or έ [1]. Regarding creep, for a given temperature and stress, for each of the parameters δ, Δχ or VEC, NICE calculates the steady-state creep rate έ [1] (attributed to intrinsic resistances to dislocation mobility [1,3]) and how the concentration of each alloying element affects the creep rate (e.g., Figure 22). Similarly, for oxidation resistance, NICE calculates the alloy weight change for each of the parameters δ, Δχ and VEC for isothermal oxidation at 800 °C or 1200 °C [1,3,19,25,26,27,43].

To summarize, NICE can calculate the composition of an alloy and the compositions of its solid solution(s) and intermetallic compound(s) (e.g., [19,25,43]), and can predict room temperature properties of an alloy and its phases (hardness, strength), Si macrosegregation, weight changes ΔW/A in isothermal oxidation and steady-state creep rate έ for a given temperature and stress (e.g., [3,19,21,25,27,28,34,43]). Owing to the relationships between έ or ΔW/A and Δχ_alloy_, δ_alloy_ or VEC_alloy_ in NICE [1,3], the latter also helps the alloy designer to understand the role/importance/contribution of each of the above parameters towards achieving the property goals and the contributions alloying additions make towards oxidation or creep properties, e.g., Figure 22 and [1]. For example, increasing the concentration of Al or Ti in the alloy increases the creep rate (Figure 22a,b), whereas increasing the concentrations of Mo and Si has the opposite effect (Figure 22c,d). Al and Ti are key additions for improving oxidation, reducing alloy density [1,3] and “balancing” properties of solid solution and intermetallic compounds; Si is important for reducing density, balancing vol.% of phases and properties of solid solution(s) and intermetallic compound(s), for improving oxidation resistance and for “balancing” the mechanical properties of the alloy. Mo is a key addition for room and high-temperature strength [3], oxidation [45], creep and alloy density [3]. Obviously, the calculations/predictions of NICE about the composition of alloys and phases and properties can be verified or not experimentally [19,20,21,25,43].

The search for metallic UHTMs that meet all three property goals simultaneously may be Sisyphean [46], but the search for creep resistance or oxidation resistance or toughness does not have a Sisyphean structure i.e., it must not be endlessly laborious or futile. According to NICE, to meet the creep goal or the oxidation goal, the alloy should have, respectively, high and low VEC_alloy_ values, whereas the opposite is the case for the parameter δ_alloy_ [1,24,26,27,28,38]. (Valence electrons also play a role regarding the ductility of solid solution RCCAs [47] and the toughness of Nb-Ti-Cr solid solutions [48]). For increased creep resistance, alloy design should aim to increase the parameter Δχ_alloy_ [1]. To put this another way, NICE recommends low VEC_alloy_ to suppress pest oxidation and improve oxidation resistance at high temperatures and high VEC_alloy_ for high-temperature strength and resistance to creep. Thus, it is unlikely that RM(Nb)ICs and RM(Nb)ICs-RHEAS/RCCAs could meet both the oxidation and creep goals simultaneously. The same was concluded by Bewlay et al. for RM(Nb)ICs [49]. Consequently, it is essential for alloy developers also to consider the development of ECs to offer environmental protection to creep-resistant metallic UHTMs.

**Figure 23:**
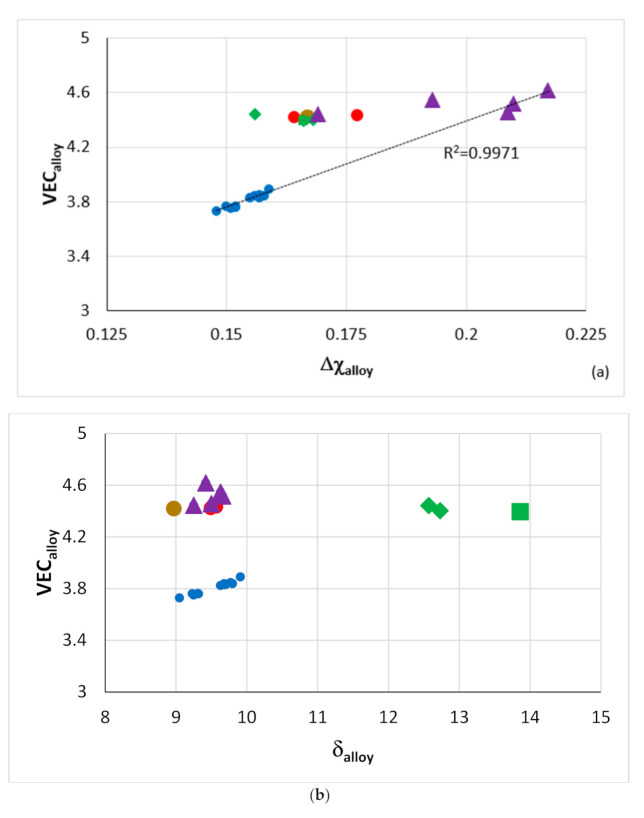

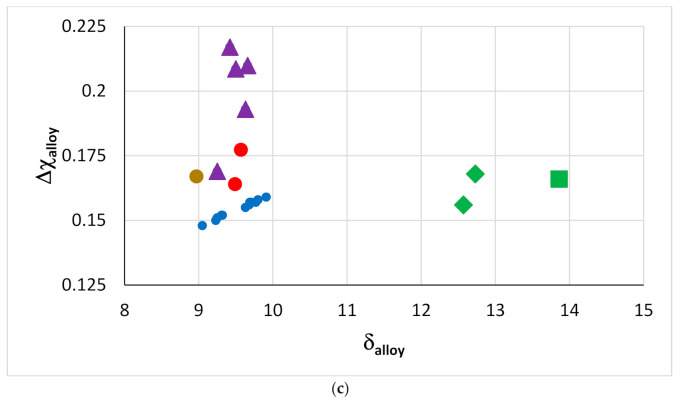
(**a**) VEC_alloy_ versus Δχ_alloy_, (**b**) VEC_alloy_ versus δ_alloy_ and (**c**) Δχ_alloy_ versus δ_alloy_ maps of RM(Nb)ICs that are also HEAs or RHEAs/RCCAs. Blue circles for HEAs of the Nb-Ti-Si-Al-Hf system, RM(Nb)ICs-RHEAs/RCCAs as follows: green with B, red with Sn, orange with Ge, purple with Ge + Sn addition. Green square for an RM(Nb)IC-RCCA with B + Sn addition.

NICE can help the alloy developer to design BC alloy(s) of ECs. Indeed, NICE has been used to design alumina forming HEAs for BCs for RM(Nb)ICs, e.g., [20,21] and to construct maps for the selection of oxidation-resistant BC HEAs or intermetallic alloys, e.g., [20,21,26]. Figure 23 shows parameter maps for HEAs of the Nb-Ti-Si-Al-Hf system and RM(Nb)ICs-RHEAs/RCCAs with/without B, or Sn or Ge or Ge + Sn or B + Sn addition, some of which belong in the Nb-Mo-W-Ti-Cr-Hf-Al-Ge-Si-Sn system (see Section 2). The B-containing alloys do not suffer from pest oxidation and scale spallation at 800 and 1200 °C [3]. Data for the weight change ΔW/A of the B-free RM(Nb)ICs-RHEAs/RCCAs and HEAs included in Figure 23 is given in Figure 16. In Figure 23, note (i) that the B-containing RM(Nb)ICs-RHEAs/RCCAs are found in distinct different areas in the VEC_alloy_ versus δ_alloy_ and Δχ_alloy_ versus δ_alloy_ maps (Figure 23b,c), (ii) that the HEAs also are in separate areas in the three maps, (iii) that the RM(Nb)ICs-RHEAs/RCCAs with Ge + Sn addition are in distinct areas in the VEC_alloy_ versus Δχ_alloy_ and Δχ_alloy_ versus δ_alloy_ maps and (iv) the linear fit of the data (R^2^ = 0.9971) for the HEAs and RM(Nb)ICs-RHEAs/RCCAs, respectively of the Nb-Ti-Si-Al-Hf and Nb-Mo-W-Ti-Cr-Hf-Al-Ge-Si-Sn systems in the VEC_alloy_ versus Δχ_alloy_ map.

The above brief discussion and [1,3] show that NICE fashions comprehensive conceptions by linking the aforementioned parameters. Each one is to some extent independent, but they are also “in line with” one another’s requirements to form a coherent whole. NICE shapes and is shaped by the idea of alloy design, and its rational procedures are ratified by the satisfactory results they deliver. We need to distinguish two main ideas (“theses”) about the relationships between data and NICE, (i) the relevant data about the alloying behavior of alloys and their phases is necessary for NICE and (ii) reliable data are a necessary “constituent element” of NICE.

NICE is (a) internally consistent, (b) responsive (to a particular property or set of properties, and to the complexities of metallic UHTMs), (c) value-relative through and through (responds to needs of the designer of metallic UHTMs and is assessed in accordance with its success in doing this), (d) self-enhancing (reminds the alloy designer that its predictions are conditional and that further work remains to be done) and (e) practical (can address specific needs of the alloy designer, makes useful and helpful contributions), and (f) gives emphasis to attention to particularity (i.e., to be exact and detailed). Users of NICE become more discriminating, more confident and more reliable in their choice of alloys that are selected for further study.

I use the “metaphor of the rope” [50] to account for the capabilities (“strength”) of NICE. A rope is made of many filaments, but not a single filament goes through the rope’s entire length. It is the way the filaments overlap and their properties that give the rope its strength. Now think of NICE as a rope and the aforementioned parameters its filaments. The capability of NICE to predict room temperature strength, hardness, isothermal oxidation behavior in the pest oxidation regime and at high temperatures, and steady-state creep rates for different temperatures and stresses, and its capacity to calculate compositions of alloys and their phases are found in (results from) the overlap of the aforementioned parameters.

## 6. Summary and Comments About Future Research

This paper considered metallic UHTMs (excluding RM(Mo)ICs) that are under development as potential replacements of Ni-based superalloys for critical applications in aeroengines and must comply with specific property goals. The approach to creating the alloy design methodology NICE [7,8,9,10,11] was revisited.

The same phases can be present in the microstructures of RM(Nb)ICs, and RHEAs and RCCAs with Nb addition, namely solid solution(s) and intermetallics(s) such as M_5_Si_3_ silicides and Laves phases. Together with Nb, the other alloying elements essentially can be the same in these metallic UHTMs and include B, Ge or Sn.

The alloying behavior of RM(Nb)ICs, RHEAs and RCCAs can be described by the same parameters, namely Δχ, δ, VEC, ΔH_mix_, ΔS_mix_, Ω. The practicality of parameter maps inspired by NICE for describing/understanding the alloying behavior and properties of alloys and their phases was demonstrated.

The relevance of NICE for the design of RM(Nb)ICs, some of which are also RHEAs and RCCAs, was highlighted. Particular emphasis was given to the alloying additions B, Ge or Sn that are suitable for RM(Nb)ICs as well as for RHEAs and RCCAs to achieve a balance of properties. To date, RHEAs and RCCAs with B, Ge or Sn additions and with A15 compounds have not been studied outside the author’s research group.

A recommendation for the development of bond coat HEAs of the Nb-Ti-Si-Al-Hf system and RM(Nb)ICs-RHEAs/RCCAs substrates of the Nb-Mo-W-Ti-Cr-Hf-Al-Ge-Si-Sn system that was made in [19,20,21] was highlighted in this paper. It was proposed that future research could investigate the effects of B, Ge or Sn additions on the properties of metallic UHTMs.

In my opinion, the present state of understanding of metallic UHTMs may reflect either the scarcity of key data, for example, how contamination by interstitial elements affects phase equilibria or mechanical properties [3,13] or information overload that may actually inhibit the conversion of information into knowledge and the reflection required to acquire understanding [3].

The creative practices of the international materials science and engineering community have stimulated technological innovation in metallic UHTMs, namely RMICs and RHEAs/RCCAs. Our thinking is formed not purely in the laboratory but also through discussions with an extensive circle of research colleagues and many hours of reading each other’s work. Collaboration between the research groups that strive for the same thing in different ways within the technological context of their time can be powerful. Efforts to match capabilities to challenges and collaboration between laboratories infused with innovative thinking should be encouraged. Researchers should take inspiration from each other’s practice. They observe, record, and act from different perspectives with the same goals and through different means—complementary perspectives perhaps, but sometimes divergent, answering to competing pulls of subjectivity and objectivity [3]. They must point to new research questions to seek a comprehensive understanding of the new materials and motivate new research for the provision of answers. The big picture emerges from the small details, from what we say to each other and what has come from this dialog.

I hope that in this paper and in the refs [1,3], I have shown by my account of NICE as much as by what it does succeed in doing, how hard and yet how exciting it is to study refractory metal alloys.

## Figures and Tables

**Table 1:** Values of the parameters ΔH_mix_, ΔS_mix_, VEC, δ, Δχ and Ω for all Nb-silicide-based alloys and their solid solutions, and for the eutectics with Nb_ss_ and Nb_5_Si_3_.

Material	Parameter					
	ΔH_mix_ (kJ/mol)	ΔS_mix_ (J/mol K)	VEC **	δ	Δχ	Ω ^+^
RM(Nb)ICs [8] and RM(Nb)ICs-RHEAs/RCCAs	−32.7 to −44.8	8.3–14.7	4.4–4.9	8.1–14.3	0.12–0.237	0.57–0.95
Solid solution in RM(Nb)ICs [7] and RM(Nb)ICs-RHEAs/RCCAs	−2 to −15.9	5.8–14.5	4.4–5.4	2.4–9.7	0.039–0.369 * gap 0.13 to 0.18	1.55–8.9
Eutectics with Nb_ss_ and Nb_5_Si_3_ [11]	−25.5 to −41.9	4.7–15	4.3–4.9	6.2–9.4	0.118–0.248 gap 0.16 to 0.18	0.38–1.35
HEAs with bcc solid solution + intermetallic(s) [7]	2.5–35	11.5–14.5	5.7–8	4.6–11	0.125–0.225	1–10
Amorphous alloys [7]	0–50	6–17.5	4–9.5	4.6–18.5	0.1–0.35	-

^+^ In [7], the letter Q was used instead of Ω for the ratio T_m_ΔS_mix_/∣ΔH_mix_∣. * In [7], the range was given as 0.039–0.331, new data in [19] changed the upper value to 0.369. ** For Nb-Ti-Si-Al-Hf HEAs (see text) 3.73 < VEC < 3.935.

## Data Availability

No new data were created or analyzed in this study.

## Appendix A

(A1) $$\delta =100\sqrt{\overset{n}{\underset{n=1}{\sum }}c_{i}{\left(1-\frac{r_{i}}{\bar{r}}\right)}^{2}}$$

(A2) $$\Delta \chi =\sqrt{\overset{n}{\underset{n=1}{\sum }}c_{i}{\left(\chi_{i}-\bar{\chi }\right)}^{2}}$$

(A3) $$\mathrm{VEC}=\overset{n}{\underset{n=1}{\sum }}c_{i}\left(\mathrm{VEC}\right)$$

(A4) $$\bar{r}=\overset{n}{\underset{n=1}{\sum }}c_{i}r_{i}$$

(A5) $$\bar{\chi }=\overset{n}{\underset{n=1}{\sum }}c_{i}\chi_{i}$$

(A6) $${\Delta H}_{\mathrm{mix}}=\overset{n}{\underset{i=1,j\neq 1}{\sum }}\Pi_{\mathrm{ij}}c_{i}c_{j}$$

(A7) $$\Pi_{\mathrm{ij}}=4{\Delta_{\mathrm{mix}}}^{\mathrm{AB}}$$

(A8) $${\Delta S}_{\mathrm{mix}}=-R\overset{n}{\underset{i=1}{\sum }}c_{i}\ln c_{i}$$

(A9) $$T_{m}=\overset{n}{\underset{i=1}{\sum }}c_{i}T_{m_{i}}$$

where c_i_, r_i_, χ_i_, (VEC)_i_ and T_mi,_ respectively, are atomic percentage, atomic radius, Pauling electronegativity, VEC and melting point of the ith element, ${\Delta_{\mathrm{mix}}}^{\mathrm{AB}}$ is the mixing enthalpy of binary liquid AB alloy and R is the gas constant.

**Table A1:** Alloys and their nominal compositions (at.%).

Alloy	Element											Ref.
	Nb	Ti	Si	Al	Cr	Hf	Ge	Sn	Mo	Ta	W	
EZ8	bal.	24	18	5	5	5	-	5	-	-	-	
JG3	bal.	24	18	5	5	-	-	-	2	-	-	[51]
JG6	bal.	24	18	5	5	5	-	5	2	-	-	[39]
JN1	bal.	24	18	5	5	5	-	-	-	-	-	[42]
JN4	bal.	20	20	-	2	2	-	5	6	-	-	
JZ3	bal.	12	18	5	5	1	5	5	-	6	2.5	[25]
JZ3+	bal.	12	18	5	5	1	5	7.5	-	6	2.5	[25]
JZ4	bal.	11.5	18	4.7	4.5	1	4.6	4.9	5	-	2	[19]
JZ5	bal.	21	18	3.7	4	0.8	4.2	4.4	6.7	-	1.2	[19]
KZ5	bal.	24	18	5	5	-	-	-	-	-	-	[31]
KZ6	bal.	24	18	5	5	-	-	-	-	6	-	[33]
OHS1	bal.	24	18	5	5	-	5	5	-	-	-	[26]
YG10	bal.	10	18	-	-	5	-	-	5	-	3	
YG11	bal.	10	18	5	-	5	-	-	5	-	3	
ZF6	bal.	24	18	5	5	-	5	-	-	-	-	[34]
ZF9	bal.	24	18	5	5	5	5	-	-	-	-	[34]

## References

[1] Tsakiropoulos P. (2018). On Nb Silicide Based Alloys: Alloy Design and Selection. Materials.

[2] Bewlay B.P., Jackson M.R., Zhao J.-C., Subramanian P.R., Mendiratta M.G., Lewandowski J.J. (2003). Ultrahigh-Temperature Nb-Silicide-Based Composites. MRS Bull..

[3] Tsakiropoulos P. (2020). Alloys for application at ultra-high temperatures: Nb-silicide in situ composites. Prog. Mater. Sci..

[4] Bewlay B.P., Jackson M.R., Gigliotti M.F.X. (2002). Niobium Silicide High Temperature In Situ Composites. Intermetallic Compounds—Principles and Practice.

[5] Jackson M.R., Bewlay B.P., Zhao J.-C. (2002). Niobium silicide based composites resistant to low temperature pesting. U.S. patent.

[6] Jackson M.R., Bewlay B.P., Briant C.L. (2002). Creep resistant Nb-silicide based two phase composites. U.S. patent.

[7] Tsakiropoulos P. (2017). On the Nb silicide based alloys: Part I—The bcc Nb solid solution. J. Alloy. Compd..

[8] Tsakiropoulos P. (2018). On Nb silicide based alloys: Part II. J. Alloy. Compd..

[9] Tsakiropoulos P. (2018). On the Alloying and Properties of Tetragonal Nb_5_Si_3_ in Nb-Silicide Based Alloys. Materials.

[10] Tsakiropoulos P. (2018). Alloying and properties of C14-NbCr_2_ and A15-Nb_3_X (X = Al, Ge, Si, Sn) in Nb-silicide based alloys. Materials.

[11] Tsakiropoulos P. (2018). Alloying and Hardness of Eutectics with Nb_ss_ and Nb_5_Si_3_ in Nb-silicide Based Alloys. Materials.

[12] Tsakiropoulos P. (2019). Alloys. U.S. patent.

[13] Senkov O.N., Miracle D.B., Chaput K.J., Couzinie J.-P. (2018). Development and exploration of refractory high entropy alloys—A review. J. Mater. Res..

[14] Berczik D.M. (1997). Method for enhancing the oxidation resistance of a molybdenum alloy, and a method of making a molybdenum alloy. US Patent.

[15] Berczik D.M. (1997). Oxidation resistant molybdenum alloy. US Patent.

[16] Schneibel J.H. (2013). High temperature strength of Mo-Mo_3_Si-Mo_5_SiB_2_ molybdenum silicides. Intermetallics.

[17] Heilmaier M., Krüge M., Saage H., Rösler J., Mukherji D., Glatzel U., Völkl R., Hüttner R., Eggler G., Somsen C. (2009). Metallic materials for structural applications beyond nickel-based superalloys. JOM.

[18] Pan K., Yang Y., Wei S., Wu H., Dong Z., Wu Y., Wang S., Zhang L., Lin J., Mao X. (2021). Oxidation behaviour of Mo-Si-B alloys at medium-to-high temperatures. J. Mater. Sci. Technol..

[19] Zhao J., Utton C., Tsakiropoulos P. (2020). On the Microstructure and Properties of Nb-18Si-6Mo-5Al-5Cr-2.5W-1Hf Nb-Silicide Based Alloys with Ge, Sn and Ti Additions (at.%). Materials.

[20] Ghadyani M., Utton C., Tsakiropoulos P. (2019). Microstructure and isothermal oxidation of the alumina scale forming Nb_1.7_Si_2.4_Ti_2.4_Al_3_Hf_0.5_ and Nb_1.3_Si_2.4_Ti_2.4_Al_3.5_Hf_0.4_ alloys. Materials.

[21] Ghadyani M., Utton C., Tsakiropoulos P. (2019). Microstructure and isothermal oxidation of the alumina scale forming Nb_1.45_Si_2.7_Ti_2.25_Al_3.25_Hf_0.35_ and Nb_1.35_Si_2.3_Ti_2.3_Al_3.7_Hf_0.35_ alloys. Materials.

[22] Einstein A. (1931). Cosmic Religion: With Other Opinions and Aphorisms.

[23] Zhang Y., Zhou Y.J., Lin J.P., Chen G.L., Liaw P.K. (2008). Solid-Solution Phase Formation Rules for Multi-component Alloys. Adv. Eng. Mater..

[24] Hernández-Negrete O., Tsakiropoulos P. (2019). On the Microstructure and Isothermal Oxidation at 800, 1200, and 1300 °C of the Al-25.5Nb-6Cr-0.5Hf (at %) Alloy. Materials.

[25] Zhao J., Utton C., Tsakiropoulos P. (2020). On the Microstructure and Properties of Nb-12Ti-18Si-6Ta-5Al-5Cr-2.5W-1Hf (at.%) Silicide-Based Alloys with Ge and Sn Additions. Materials.

[26] Hernández-Negrete O., Tsakiropoulos P. (2020). On the Microstructure and Isothermal Oxidation at 800 and 1200 °C of the Nb-24Ti-18Si-5Al-5Cr-5Ge-5Sn (at.%) Silicide-Based Alloy. Materials.

[27] Li Z., Tsakiropoulos P. (2019). The Effect of Ge Addition on the Oxidation of Nb-24Ti-18Si Silicide Based Alloys. Materials.

[28] Hernández-Negrete O., Tsakiropoulos P. (2019). On the Microstructure and Isothermal Oxidation of Silica and Alumina Scale Forming Si-23Fe-15Cr-15Ti-1Nb and Si-25Nb-5Al-5Cr-5Ti (at.%) Silicide Alloys. Materials.

[29] Hernández-Negrete O., Tsakiropoulos P. (2019). On the Microstructure and Isothermal Oxidation of the Si-22Fe-12Cr-12Al-10Ti-5Nb (at.%) Alloy. Materials.

[30] Guo S., Liu C.T. (2011). Phase stability in high entropy alloys: Formation of solid-solution phase or amorphous phase. Prog. Nat. Sci. Mater. Int..

[31] Zelenitsas K., Tsakiropoulos P. (2005). Study of the role of Cr and Al additions in the microstructure of Nb-Ti-Si in situ composites. Intermetallics.

[32] Grammenos I., Tsakiropoulos P. (2011). Study of the role of Hf, Mo and W additions in the microstructure of Nb–20Si silicide based alloys. Intermetallics.

[33] Zelenitsas K., Tsakiropoulos P. (2006). Study of the role of Ta and Cr additions in the microstructure of Nb-Ti-Si-Al in situ composites. Intermetallics.

[34] Li Z., Tsakiropoulos P. (2019). On The Microstructures and Hardness of The Nb-24Ti-18Si-5Al-5Cr-5Ge and Nb-24Ti-18Si-5Al-5Cr-5Ge-5Hf (at.%) Silicide Based Alloys. Materials.

[35] Pugh S. (1954). XCII. Relations between the elastic moduli and the plastic properties of polycrystalline pure metals. Lond. Edinburgh Dublin Philos. Mag. J. Sci..

[36] Li Z., Tsakiropoulos P. (2011). Study of the effect of Ti and Ge in the microstructure of Nb-24Ti-18Si-5Ge in situ composite. Intermetallics.

[37] Li Z., Tsakiropoulos P. (2012). Study of the effect of Cr and Ti additions in the microstructure of Nb–18Si–5Ge based in-situ composites. Intermetallics.

[38] Xu Z., Utton C., Tsakiropoulos P. (2020). A Study of the Effect of 5 at.% Sn on the Micro-Structure and Isothermal Oxidation at 800 and 1200 °C of Nb-24Ti-18Si Based Alloys with Al and/or Cr Additions. Materials.

[39] Geng J., Tsakiropoulos P., Shao G. (2007). A study of the effects of Hf and Sn additions on the microstructure of Nb_ss_/Nb_5_Si_3_ based in situ composites. Intermetallics.

[40] Tsakiropoulos P., Zelenitsas K., Vellios N. (2011). Study of the effect of Al, Cr and Sn additions on the microstructure and properties of Nb silicide based alloys. MRS Proc..

[41] McCaughey C., Tsakiropoulos P. (2018). Type of Primary Nb_5_Si_3_ and Precipitation of Nb_ss_ in αNb_5_Si_3_ in a Nb-8.3Ti-21.1Si-5.4Mo-4W-0.7Hf (at.%) Near Eutectic Nb-Silicide-Based Alloy. Materials.

[42] Nelson J., Ghadyani M., Utton C., Tsakiropoulos P. (2018). A Study of the Effects of Al, Cr, Hf, and Ti Additions on the Microstructure and Oxidation of Nb-24Ti-18Si Silicide Based Alloys. Materials.

[43] Zhao J., Utton C., Tsakiropoulos P. (2020). On the microstructure and properties of Nb-12Ti 18Si-6Ta-2.5W-1Hf (at.%) silicide based alloys with Ge and Sn additions. Materials.

[44] Tsakiropoulos P. (2014). On the macrosegregation of silicon in niobium silicide based alloys. Intermetallics.

[45] Geng J., Tsakiropoulos P., Shao G. (2006). Oxidation of Nb–Si–Cr–Al in situ composites with Mo, Ti and Hf additions. Mater. Sci. Eng. A.

[46] Sisyphus. https://www.britannica.com/topic/Sisyphus.

[47] Sheikh S., Shafeie S., Hu Q., Ahlström J., Persson C., Veselý J., Zýka J., Klement U., Guo S. (2016). Alloy design for intrinsically ductile refractory high-entropy alloys. J. Appl. Phys..

[48] Davidson D.L., Chan K.S., Anton D.L. (1996). The effects on fracture toughness of ductile-phase composition and morphology in Nb-Cr-Ti and Nb-Siin situ composites. Met. Mater. Trans. A.

[49] Bewlay B.P., Whiting P.W., Davis A.W., Briant C.L. (1998). Creep Mechanisms in Niobium-Silicide Based In-Situ Composites. MRS Proc..

[50] Wittgenstein L. (1953). Philosophical Investigations.

[51] Geng J., Tsakiropoulos P., Shao G. (2006). The effects of Ti and Mo additions on the microstructure of Nb-silicide based in situ composites. Intermetallics.

